# Isoniazid-derived ionic liquids for enhanced C-steels surface finishing via electrochemistry, supported by Monte Carlo, mechanistic study, theoretical, and docking methodology

**DOI:** 10.1038/s41598-026-55745-w

**Published:** 2026-06-16

**Authors:** Amira Hossam Eldin Moustafa, Ali Atya Harfoush, Hanaa Hammam Abdel-Rahman, Mohamed Hagar, Menna Mamdouh

**Affiliations:** Chemistry Department, Faculty of Science, Alexandria University, Ibrahemia, P.O. 426, Alexandria, 21321 Egypt

**Keywords:** Surface electropolishing, Isoniazid ionic liquid, Galvanostatic analysis, Computational modeling, Docking study, SRB inhibition, SEM, EDX, XPS, AFM, MC, Chemistry, Materials science

## Abstract

Ecological concerns, stringent environmental laws, and the ongoing need for innovation motivated this investigation into improved metal-finishing techniques. Proper polishing methods can improve qualities such as corrosion and wear resistance by drastically reducing surface roughness and the coefficient of friction. However, traditional polishing methods have certain limitations that cause them to consume the metal. In this study, we introduced a newly *aromatic pyridinium salt based isoniazid hybrid compounds* (**APyHC**) as eco-friendly synthesized ionic liquids (ILs) to diminish the electrochemical dissolution rates (Electropolishing EP) of carbon steels (CSs) improving surface protection and demonstrate inhibition efficiencies (%IE) mostly 80% at relatively low concentration 5.93 × 10^− 5^ M in 8M H_3_PO_4_, offering a sustainable alternative to harmful inhibitors. To comprehensively evaluate the inhibitor’s performance, we employed Galvanostatic mass-transfer analysis over a temperature range of 293–308 K. This test revealed a significant improvement in surface finish. The kinetic and thermodynamic coefficient values were calculated. The results show that increasing **APyHC** concentration enhanced inhibition efficiency, whereas rising temperature reduced it, confirming a temperature-sensitive process. Adsorption followed a mixed physicochemical mechanism obeying the El-Awady model (*R² = 0.99* at 298 K). Thermodynamic parameters confirmed endothermic, spontaneous adsorption, with *E*_*a*_ below 80 kJ/mol, positive *ΔH°*, and *ΔG°* ranging from − 37.40 to -39.77 kJ/mol. The surface characteristics of CSs, as measured by composition (*EDX*, *XPS*), morphology (*SEM*), and topography (*AFM*), confirmed the presence of surface shielding. In line with experimental techniques, theoretical insights, such as *DFT* calculations and quantum-based *Monte Carlo simulations* (*MC*), were integrated with experiments to clarify reactivity, adsorption mechanisms at the atomistic scale, deepening understanding of inhibition and supporting a sustainable strategy for designing green ionic-liquid-based protection systems. Additionally, docking simulations examined **APyHC**’s biological affinity for the sulfate-reducing bacteria (**SRB**) protein, evaluating its potential as a dual-purpose inhibitor of CSs dissolution in harsh media.

**Supplementary Information:**

The online version contains supplementary material available at 10.1038/s41598-026-55745-w.

## Introduction

The metal finishing industry is adopting cleaner methods due to stricter regulations, recognizing that surface quality dictates metal performance and lifespan. These preventive actions have a positive impact on both the environment and these companies’ ability to compete. This is because fewer raw materials are used, less energy is consumed, and contaminated materials and outflows are reduced, thereby reducing the risks of environmental contamination^1^. Using a variety of cleaner production techniques is challenging, though. Furthermore, this might have a detrimental impact, particularly when the whole picture is considered, underscoring the need for efficient decision aids in this industry^2^.

Industrial demand has driven efforts to evaluate surface roughness due to its impact on corrosion resistance. Polishing serves a dual purpose: as a final treatment to reduce roughness and improve durability, and as a pretreatment to enhance coating adhesion, resulting in stronger interfacial bonding and improved product longevity^3,4^.

Therefore, Electropolishing (EP) is an electrochemical finishing process that smooths metal surfaces by selectively dissolving microscopic peaks. Still, it requires a controlled current density to prevent oxygen bubble formation, which can cause pitting and reduce corrosion resistance^5^. This is a major problem for carbon steel (used in construction, automotive, and pipelines), as the acidic EP process makes it susceptible to damaging pitting rather than protecting it^6,7^.

Specialized corrosion inhibitors are crucial in electro-polishing to prevent pitting and gas evolution^8^. Highly effective organic inhibitors work by adsorbing onto the metal surface using heteroatoms (N, S, O), polar groups (-NH₂, -COOH, -OH), and π-electrons as active adsorption sites^9,10^. This creates a stable insulating film that blocks corrosion sites, significantly lowering corrosion rates even at low concentrations and protecting the metal^11,12^.

For developing effective organic inhibitors, cyclic imide derivatives have versatile applications, including use as metal inhibitors, resin solvents, and antimicrobial agents^13^. Isonicotinohydrazide derivatives have been the subject of considerable research, which consistently confirms their potent antibacterial efficacy. Notwithstanding their established value in biological contexts, their potential utility as corrosion inhibitors remains largely unexplored^14–17^. The corrosion inhibition potential of pyridine-based compounds has been the focus of considerable research. In chemical synthesis, pyridine serves as an organic base, forming pyridinium salts that participate in various acid-base reactions. These salts are commercially valuable and find use in sectors such as pharmaceuticals, cosmetics, and industrial catalysis^18^. In their liquid state at ambient temperature, they are classified as pyridinium ionic liquids. Their biological applications include drug and gene delivery, owing to their properties, such as their interactions with biomembranes and their DNA-binding capabilities. A key mechanism of their effectiveness as corrosion inhibitors is anticipated to be intermolecular synergism, in which the large cationic and anionic groups enhance adsorption onto metallic substrates.

Therefore, in line with what was mentioned last, for metal electropolishing, ionic liquids (ILs) offer a sustainable and less hazardous alternative to conventional mechanical surface treatments^19^. Critically, these same properties make ionic liquids environmentally benign, with high thermal and chemical stability, and also drive their exceptional corrosion-inhibition efficacy, ensuring resilience in aggressive environments^20,21^. Concurrently, non-volatility ensures persistent surface coverage without evaporative loss, a key requirement for long-term protection. While high polarity and tunable design enable durable protective film formation that collectively blocks corrosive agents, minimizing environmental impact and aligning with green chemistry principles^22,23^.

Molecular docking is a key computational method for designing antibacterial compounds and anodic dissolution inhibitors. It simulates binding interactions with biological targets to optimize antibacterial activity and assess effects on corrosion (either inhibition or unintentional promotion), aiding development of novel anti-corrosion strategies. Additionally, Monte Carlo (MC) simulations predict metal-inhibitor interactions and adsorption energy at the adsorbate-substrate interface^24,25^.

Given the aforementioned reasoning, the current study seeks to improve the CSs’ surface properties to achieve a smooth, shiny microstructure and enhanced corrosion resistance, thereby reducing metal dissolution and surface degradation. So, we introduced this by highlighting the importance of imide, Isonicotinohydrazide, and pyridinium salts in developing two novel hybrid compounds, eco-friendly **APyHC**, as high-performance inhibitors for the electropolishing of CSs in aggressive 8 M phosphoric acid. Based on the unique physical properties of ILs and on understanding the EP mechanism, our primary objective was to rigorously quantify their inhibition efficacy and elucidate the associated adsorption thermodynamics across a range of concentrations and temperatures. The resulting surface improvements were analyzed using advanced characterization techniques, including scanning electron microscopy with energy-dispersive X-ray spectroscopy (SEM-EDX) and atomic force microscopy (AFM), to assess morphology and homogeneity. Furthermore, X-ray photoelectron spectroscopy (XPS) was employed to characterize the elemental composition and confirm the successful formation of a protective inhibitory film on the CSs.

Furthermore, this study seeks to connect the experimentally observed inhibitory effects of **APyHC** on CSs with computational analyses using Density Functional Theory (DFT). These computations elucidated the electronic structures of the inhibitors, identified key reactive sites, and quantified adsorption energetics, thereby establishing a fundamental structure-performance relationship of Fukui indices, Molecular electrostatic potential, and Electron Localization Function (ELF). We also utilize molecular docking simulations to evaluate the compound’s antibacterial activity against Sulfate-Reducing Bacteria (SRB). By integrating these methods, we establish a comprehensive and predictive strategy for designing new multifunctional pyridinium-based compounds **APyHC**, which hold considerable promise for both industrial and biomedical applications. The theoretical approach analyzes not only the basic quantum parameters but also integrates spatial visualization of inhibitor molecule adsorption onto the Fe(110) surface via Monte Carlo simulation (MC), linking molecular orientation to adsorption energy. The combination of extreme test conditions and in-depth theoretical analysis makes this study an original contribution to developing the best surface protection and demonstrating greenish inhibitors.

## Materials and methods

### Preparation of ILs

A mixture of N-(1,3-dioxoisoindolin-2-yl)isonicotinamide (3) (0.01 mol) and the appropriate trifluoromethyl benzyl bromide 4a-b (0.01 mol): 1-(bromomethyl)−2-(trifluoromethyl)benzene (4a) or 1-(bromomethyl)−4-(trifluoromethyl)benzene (4b) in 15 ml Acetonitrile. The reaction mixture was heated under reflux for 12 h, and the excess solvent was removed. The obtained solid was filtered off and crystallized from ethanol to afford the desired products. The results obtained from characterization are reported in the following Table 1.. The material, the characterization equipment, and the resulting Figs. S1-S6 are noted in the Supplementary File.

### Sample preparation and test solution

All electrochemical measurements were performed using CSs working electrodes with a 10 cm² exposed surface area. A rigorous surface preparation protocol, applied before each experiment, consisted of sequential mirror-polishing with fine-grit emery paper, degreasing with acetone, rinsing with distilled water, and final air-drying at ambient temperature. Elemental analysis using a JEOL JSM-IT200 apparatus confirmed the material composition (wt%) as: Fe (99.15%), C (0.14%), Mn (0.56%), P (0.03%), S (0.02%), Al (0.03%), Si (0.02%), Cr (0.01%), V (0.01%), and Ni (0.01%)^26,27^.

The test solution was an 8 M phosphoric acid (H₃PO₄) solution, formulated by diluting analytical-grade reagent (85% w/w, Fisher Chemicals Ltd.) with distilled water. The inhibition performance of ***o***-**APyHC** and ***p***-**APyHC** was assessed in a 100 mL electrochemical cell across a concentration range of (0.494–5.928) × 10⁻⁵ M. These test solutions were prepared from a 1.976 × 10⁻³ M stock solution in distilled water. The limiting current (Iₗ_i_ₘ) was determined for each inhibitor concentration at four different temperatures: 293, 298, 303, and 308 K. This temperature range was deliberately selected to preserve the stability of the ionic liquids, thereby enabling accurate investigation of corrosion processes without interference from solvent decomposition. Moreover, it covers both ambient conditions (20–25 °C) and moderately elevated temperatures (up to 35–40 °C), making it highly representative of practical industrial environments such as pipelines, chemical processing facilities, heat exchangers, and aerospace systems. All measurements were performed in triplicate, with reported values as averages showing excellent reproducibility and a maximum experimental error of ± 0.2%.

### Galvanostatic polarization measurements

Galvanostatic polarization was employed to assess inhibitor efficiency, leveraging its well-documented reliability, accuracy, and experimental simplicity in accordance with standardized literature procedures.

**Table 1.** FT-IR, ^1^H NMR, and ^13^C NMR data for the synthesized ionic liquids (**APyHC**).

Compound		
Molecular Formula (IL)	C_22_H_15_BrF_3_N_3_O_3_(**o-APyHC**)	C_22_H_15_BrF_3_N_3_O_3_ (**p-APyHC**)
Color	yellowish-white crystals	off-white crystals
Yield	82%	84%
FT-IR ν_max_ (cm^−1^, KBr)	3389 (NH), 2891 (CH_2_) sp^3^-stretch, 1787, 1744 (C=O, acid anhydride), 1699(C=O, secondary amide) were observed as strong bands (S1).	3404 (NH), 2855 (CH2) sp3-stretch, 1790, 1740 (C=O, acid anhydride), 1705 (C=O, secondary amide) were observed as strong bands (S4).
^1^H NMR; δ (ppm) (500 MHz, DMSO-d6)	12.35 (s, 1H, NH), 9.35 (d, J = 6.0 Hz, 2H, Ar-H), 8.46 (d, J = 6.0 Hz, 2H, Ar-H), 8.20 (t, J = 4.5 Hz, 2H, Ar-H), 7.96 (dd, J = 4.5 Hz, J = 2.0 Hz, 2H, Ar-H), 7.91 (d, J = 7.5 Hz, 1H, Ar-H), 7.72 (t, J = 7.5 Hz, 1H, Ar-H), 7.67 (t, J = 7.5 Hz, 1H, Ar-H), 7.29 (d, J = 7.5 Hz, 1H, Ar-H), 6.23 (s, 2H, CH_2_) (S2).	12.25 (s, 1H, NH), 9.44 (d, J = 6.0 Hz, 2H, Ar-H), 8.59 (d, J = 6.0 Hz, 2H, Ar-H), 8.01 (dd, J = 5.0 Hz, J = 3.0 Hz, 2H, Ar-H), 7.96 (dd, J = 5.0 Hz, J = 3.0 Hz, 2H, Ar-H), 7.82 (d, J = 8.5 Hz, 1H, Ar-H), 7.74 (d, J = 8.5 Hz, 1H, Ar-H), 6.06 (s, 2H, CH_2_) (S5).
^13^C NMR (125 MHz, DMSO-d6)	165.2, 162.0, 147.7, 145.6, 136.2, 134.2, 131.7, 131.1, 130.5, 129.9, 127.5, 127.3, 124.7(Ar-C), 60.9 (CH_2_) (S3).	165.2, 162.1, 147.3, 145.3, 136.2, 130.3, 129.9, 127.4, 126.6, 126.5, 126.2, 124.6, 123.4(Ar-C), 63.4 (CH_2_) (S6).
CHN (%) (Found)	C=52.28, H=3.04,N=8.38	C=52.28, H=3.04, N=8.38
CHN (%) (Calculated)	C=52.19, H=2.99, N=8.30	C=52.19, H=2.99, N=8.30

The surface coverage (θ) of the electropolished steel and the inhibition efficiency (% IE) were calculated according to Eqs^8,28^:

1 $$\:{\uptheta\:}=\raisebox{1ex}{${I}_{\mathrm{l}\mathrm{i}\mathrm{m}\boldsymbol{B}\boldsymbol{l}\boldsymbol{a}\boldsymbol{n}\boldsymbol{k}}-{I}_{\mathrm{l}\mathrm{i}\mathrm{m}\boldsymbol{A}\boldsymbol{P}\boldsymbol{y}\boldsymbol{H}\boldsymbol{C}}$}\!\left/\:\!\raisebox{-1ex}{$\:{I}_{\mathrm{l}\mathrm{i}\mathrm{m}\boldsymbol{B}\boldsymbol{l}\boldsymbol{a}\boldsymbol{n}\boldsymbol{k}}$}\right.$$

2 $$\% IE{\text{ }} = {\text{ }}\theta \times 100$$

Where *I*_*lim****blank***_ is the limiting current in the absence of inhibitors, and *I*_*lim****APyHC***_ is the limiting current in the presence of it.

### Topography analysis

Surface analysis was conducted on polished CSs specimens (1.0 cm × 1.0 cm × 0.3 cm). These were exposed to 8 M H₃PO₄ under different conditions of concentration and temperatures, testing both in the attendance and nonattendance of the **APyHC**. After air-drying and storage in a desiccator, the samples were characterized. This included capturing two-dimensional SEM micrographs with a JEOL JSM-IT200, with a coupled EDX detector for quantitative analysis of film chemical composition. All micrographs of the carbon steel coupons were taken at a magnification of 5000. Surface topography and roughness were further examined via AFM using an Auto Probe CP-Research system (ThermoMicroscope), operating in contact mode with a Bruker MLCT Silicon Nitride probe. Topographic images were captured over a 25 × 25 μm² area with a scan rate of 1 Hz and a resolution of 256 × 256 pixels. Data acquisition was managed with ProScan 1.8 software, while image analysis was performed with IP 2.1 software.

X-ray photoelectron spectroscopy (XPS) was carried out using a Thermo Fisher Scientific K-Alpha spectrometer. The analysis utilized monochromatic Al Kα radiation over a binding energy range of −10 to 1350 eV under an ultra-high vacuum of 10⁻⁹ mbar. Survey and high-resolution spectra were acquired with pass energies of 200 eV and 50 eV, respectively, using a 400 μm analysis spot. All binding energies were referenced to the adventitious C 1 s peak.

### Quantum chemical calculations

The molecular structures of the investigated **APyHC** were constructed in GaussView and subsequently optimized. All calculations were performed using Density Functional Theory (DFT) within the Gaussian 09 software package, employing the B3LYP functional and the 6-311G(d, p) basis set. This computational methodology is well-established in corrosion science for its accuracy in predicting molecular properties^12^. The molecular dynamics simulations were performed using the Adsorption Locator module with the COMPASS force field. The Fe (110) surface was selected as the adsorption substrate owing to its high thermodynamic stability. The simulation box dimensions were set to 45 × 60 × 45 Å³ under periodic boundary conditions to realistically simulate the corrosive acidic environment containing inhibitor molecules, water molecules, and H_3_PO_4_ species. Simulated annealing calculations were performed with 20 annealing cycles of 2500 steps each, under automated temperature control. Geometry optimization was performed before the simulations to minimize the system’s total energy. The adsorption configurations and adsorption energies were subsequently extracted from the equilibrium trajectories to evaluate the interaction strength between the inhibitor molecules and the *Fe* surface. Key global reactivity descriptors were calculated using equations in the Supplementary File.

### Molecular docking simulation

Molecular docking simulations were performed to investigate the binding mode and inhibitory potential of the synthesized ligands *o-APyHC* and *p-APyHC* toward the active site of ribosyltransferase from bacterial origin (PDB ID: 3GEY). Docking studies were carried out using AutoDock 4.0 with the Lamarckian Genetic Algorithm (LGA) under default parameters. All ligands were energy-minimized prior to docking, and the grid box was positioned to fully enclose the enzyme’s catalytic pocket. Several conformers were generated for each ligand and ranked according to the AutoDock scoring function. The most stable conformation was selected based on its lowest binding free energy (ΔG), intermolecular interaction energy, internal torsional energy, and predicted inhibition constant (K_i_). Protein–ligand interactions were further analyzed using Discovery Studio, with emphasis on hydrogen bonding, halogen interactions, π–cation contacts, π–π stacking, and hydrophobic interactions, providing a comprehensive evaluation of the stability and biological relevance of the docked complexes.

## Results and discussion

### Synthesis and structural characterization

**Scheme 1:**
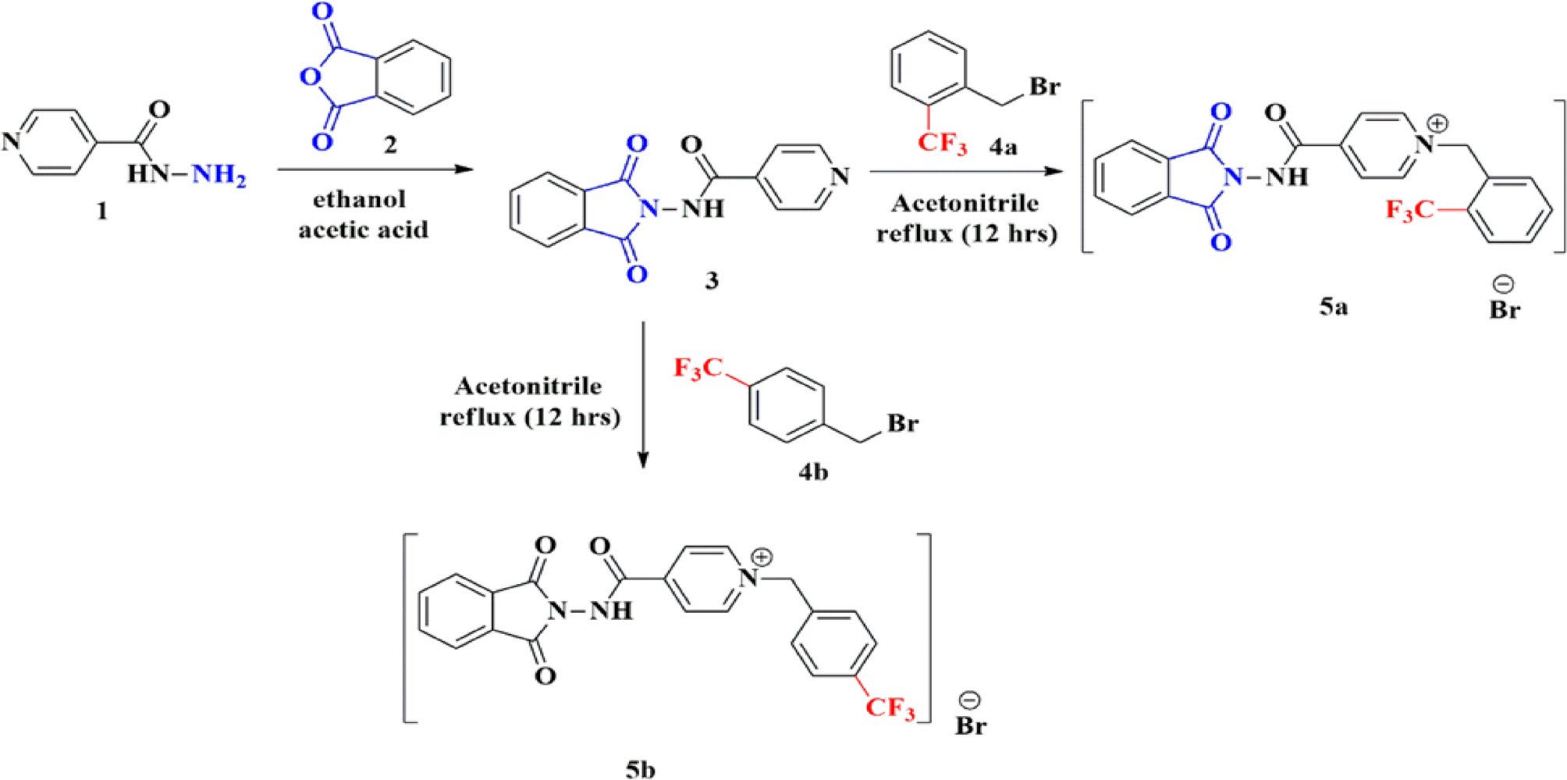
Synthesis of new isoniazid-based ionic liquid derivatives.

Herein, our work focused on the preparation of a new ionic liquid based on the isoniazid moiety. As adopted in Scheme 1, the condensation reaction of isonicotinohydrazide (1) and phthalic anhydride (2) in ethanol as solvent and drops of glacial acetic acid to afford N-(1,3-dioxoisoindolin-2-yl)isonicotinamide (3) in a high yield^29^.

Two pyridinium bromide ionic liquid derivatives, 5a and were synthesized *via* reaction of N-(1,3-dioxoisoindolin-2-yl)isonicotinamide (3) with different trifluoromethyl benzyl bromide, namely 1-(bromomethyl)−2-(trifluoromethyl)benzene (4a) or 1-(bromomethyl)−4-(trifluoromethyl)benzene (4b) in acetonitrile as solvent. The structure of the synthesized compounds 5a and 5c was confirmed using spectroscopic data. Their FT-IR spectrum showed a sharp band of the C = O stretching vibration of the secondary amide at 1699 and 1705 cm^− 1^, and their 13 C-NMR spectrum showed a peak of CH_2_ at 60.9 and 63.4 ppm for compounds 5a and 5b, respectively. Moreover, for compound 5a, its^1^H-NMR spectrum showed two triplet peaks in the aromatic range at 7.67 and 7.72 ppm, confirming the presence of a trifluoromethyl group in the ortho position. In contrast, for compound 5b, it appears as two doublet peaks at 7.82 and 7.74 ppm, confirming the presence of a trifluoromethyl group in the para position (Table 1).

### Galvanostatic polarization studies

The Galvanostatic polarization (GP) method was employed to evaluate the influence and kinetics of **APyHC**on the anodic dissolution of CSs during EP. This approach was chosen due to its simplicity, low cost, and numerous advantages over alternative methods^30,28^. Additionally, it offers a practical means to determine solid/liquid mass transfer rates, such as measuring electro-recovery kinetics under diffusion-limited conditions. The technique provided important information on the inhibition mechanism, both in the absence and with different concentrations of the **APyHC** inhibitor, across temperatures ranging from 293 to 308 K in 8 M H₃PO₄.

**Fig. 1:**
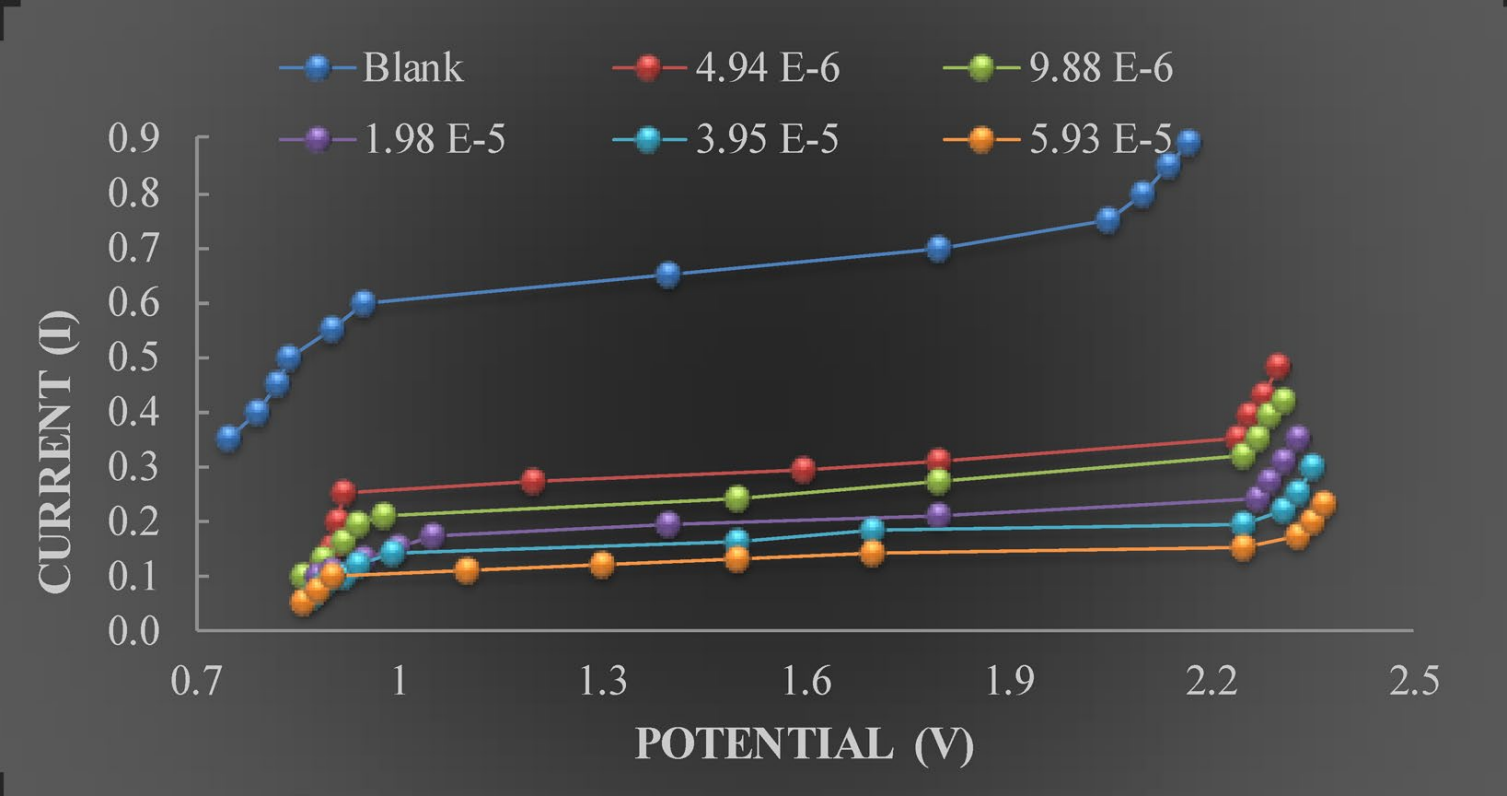
Schematic of the current/voltage profiles during the galvanostatic polarization experiments for *p-APyHC* at 293 K.

As shown in Table 2, increasing the **APyHC** concentration results in a more negative EP rate and a reduction in the limiting current. This occurs because inhibitor molecules bind to active sites on the CS surface, slowing the EP process. Polarization curves exhibit a plateau both with and without the inhibitor (Fig. 1), suggesting the development of a more effective protective oxide layer on the metal. The shape of the plateau changes only marginally at higher inhibitor concentrations. These findings indicate that the inhibitor restricts anodic dissolution, with inhibition efficiency increasing significantly as concentration increases, consistent with an adsorption-based mechanism in which ionic liquid (IL) molecules bind to the steel surface. Maximum inhibition efficiencies reached 80.99% for **p-APyHC** and 74.67% for *o-APyHC* (at 5.93 × 10⁻⁵ M), confirming the effectiveness of these ILs as EP inhibitors in acidic media. The percentage %IE (Table 2) and surface coverage were calculated using Eqs. (1) and (2), respectively. Moreover elevating the temperature from 293 to 308, resulting in a shift in limiting current values followed by a decrease for the corresponding %IE as holding the inhibitor concentration constant as in Table 2.

**Table 2:** Depicted the variation in EP rates as I_Lim_, and (% IE) with different **APyHC** dosages in 8M H_3_PO_4_ at different temperatures.

ILs		C x 10^5^ (M)	293 K		298K		303 K		308 K	
			I_Lim_(A)	% IE	I_Lim_(A)	% IE	I_Lim_(A)	% IE	I_Lim_(A)	% IE
*p*-**APyHC**		Blank	0.75±0.18	0.00	0.80±0.21	0.00	0.86±0.23	0.00	0.95±0.27	0.00
		0.49	0.35±0.02	53.33	0.42±0.03	47.50	0.52±0.04	39.53	0.62±0.16	34.74
		0.99	0.32±0.01	57.33	0.37±0.02	53.75	0.42±0.03	51.16	0.52±0.12	45.26
		1.98	0.24±0.02	68.00	0.30±0.01	62.50	0.36±0.02	58.14	0.45±0.09	52.63
		3.95	0.19±0.01	74.67	0.28±0.01	65.00	0.33±0.02	61.63	0.41±0.05	56.84
		5.93	0.15±0.01	80.00	0.25±0.01	68.75	0.32±0.02	62.79	0.39±0.03	58.95
*o*-**APyHC**		Blank	0.75±0.18	0.00	0.80±0.21	0.00	0.86±0.23	0.00	0.95±0.27	0.00
		0.49	0.40±0.05	46.67	0.44±0.03	45.00	0.55±0.04	36.05	0.68±0.18	28.42
		0.99	0.35±0.02	53.33	0.41±0.03	48.75	0.47±0.04	45.35	0.54±0.15	43.16
		1.98	0.27±0.02	64.00	0.32±0.03	60.00	0.41±0.02	52.33	0.49±0.10	48.42
		3.95	0.23±0.01	69.33	0.30±0.02	62.50	0.37±0.02	56.98	0.42±0.08	55.79
		5.93	0.19±0.01	74.67	0.26±0.01	67.50	0.33±0.01	61.63	0.40±0.05	57.89

### Temperature impact and thermodynamic activation properties

To evaluate the durability of the adsorbed ILs film on the CSs’surface and to calculate the thermodynamic activation parameters for the EP process in the aggressive medium, Galvanostatic measurements were performed over a temperature range of 293–308 K, both with and without the optimal concentrations of **APyHC**. Based on Table 2, dissolution rates were significantly enhanced at higher temperatures, with a maximum observed at 308 K in the uninhibited solution. This increase in the EP rate at elevated temperatures is caused by faster molecular interactions, which change how the CSs surfaces interact with the acidic solution. Generally, higher temperatures promote anodic reactions, thereby affecting the kinetics of the EP process^27,31^.

**Fig. 2:**
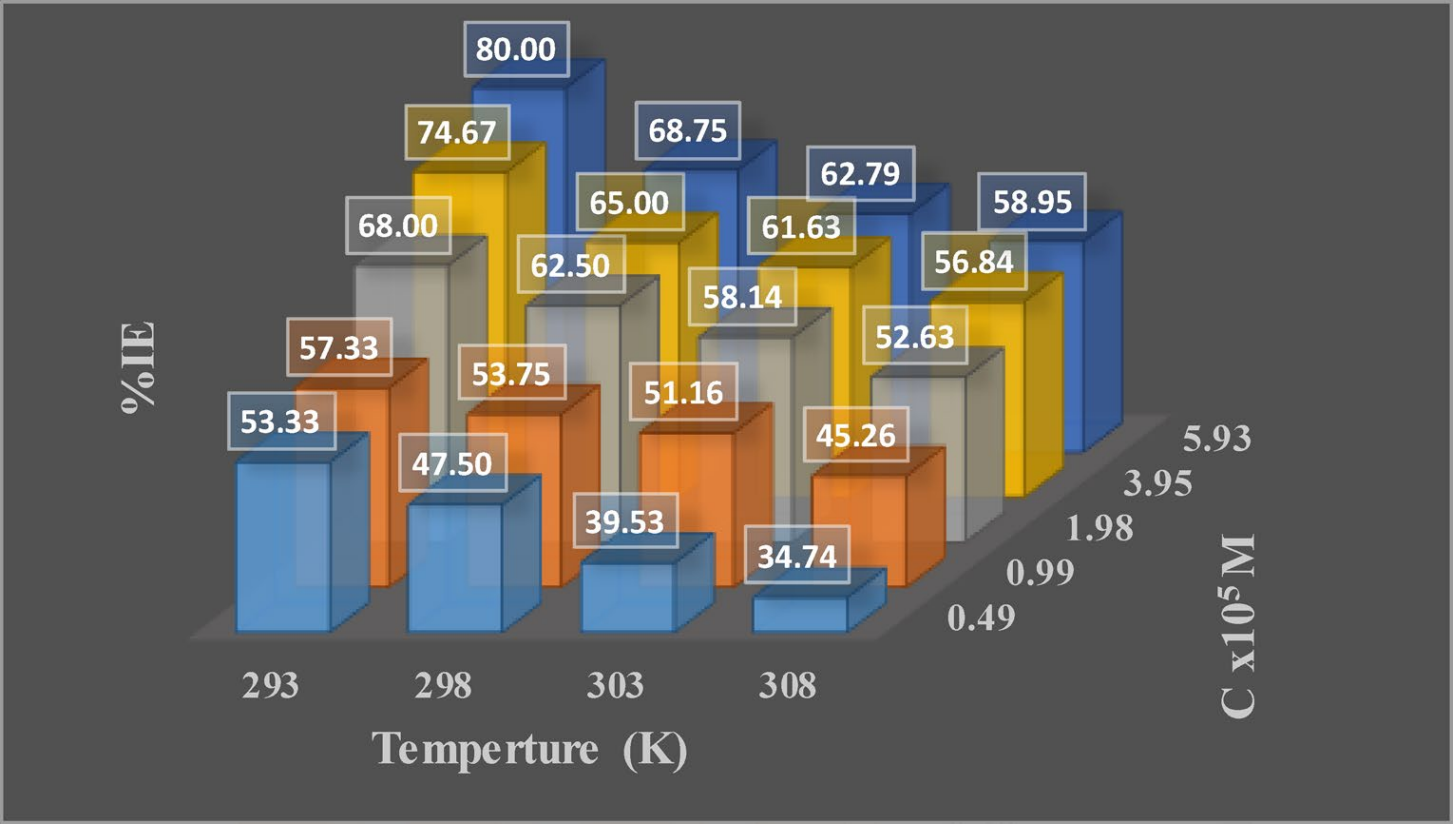
Figure1 A bar chart illustrating the relative performance of the *p-***APyHC** at different temperatures.

At a constant **APyHC** concentration, the %IE decreased with increasing temperature, as illustrated in Fig. 2. This decline is principally due to the increase in thermal motion, which shifts the adsorption-desorption equilibrium toward desorption, explaining the decrease in **APyHC**’s inhibitory performance, which reduces the integrity (weakening) of the protective layer. This feature is identified as physisorption, relying on weak electrostatic bonds that heat can easily disrupt^32^. Even with lower efficiency at elevated temperatures, substantial dissolution inhibition was still achieved. This reinforces the potential of these ILs.

**Fig. 3:**
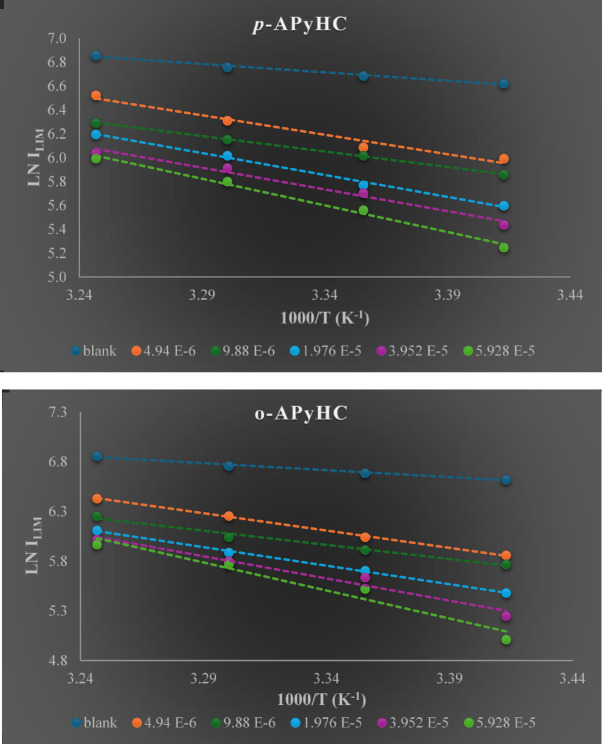
Figure1 Arrhenius plots depicting the EP of CSs in 8M H_3_PO_4_, with and without various concentrations of **APyHC**.

**Fig. 4:**
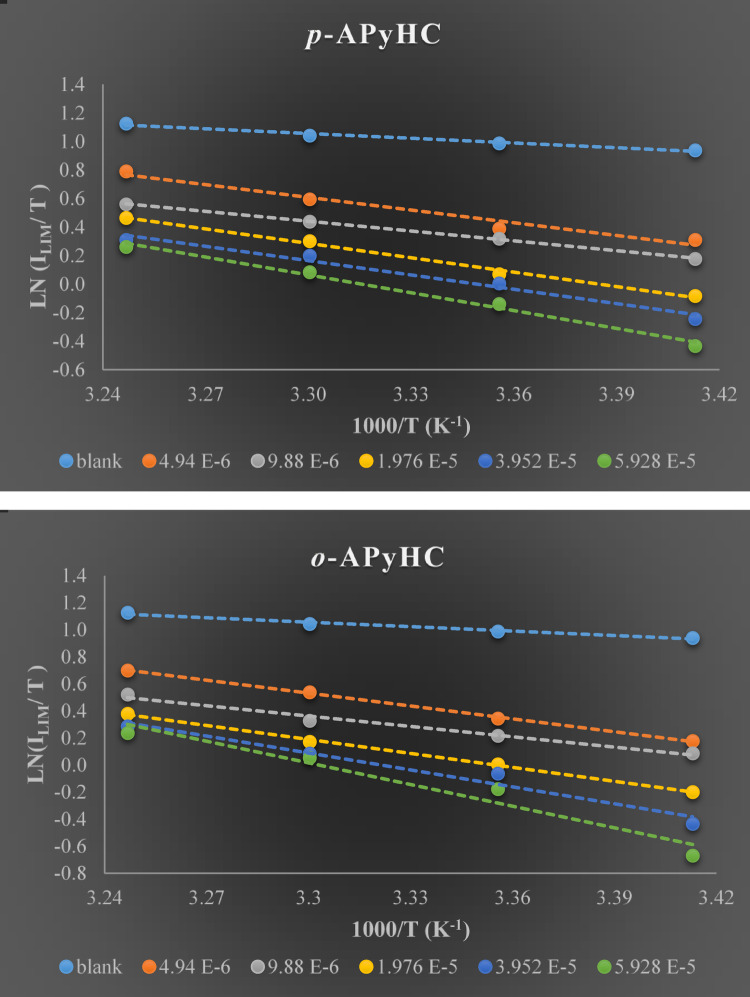
Transition plots comparison for CSs in 8M H_3_PO_4_, tested both with and without various concentrations of **APyHC**.

The inhibition mechanism is further elucidated by changes in activation energy (*E*_*a*_). An observed decline in inhibition efficiency at elevated temperatures, coupled with a rise in *E*_*a*_. The activation energies were determined through application of the Arrhenius Eq^33^:

3 $$\ln {\text{ }}I_{{Lim}} = \ln A - ~\:\:\frac{{\:\:E_{a}}}{{RT}}~~~~~~~~~~$$

where *A* is the pre-exponential factor, *R* is the universal gas constant (8.314 J mol⁻¹ K⁻¹), and *T* is the thermodynamic temperature (293–308 K). Linear Arrhenius plots (Fig. 3) facilitated *E*_*a*_ calculations. Table 3 summarizes the activation parameters for the EP process of CSs in acid. The rise in activation energy (*E*_*a*_) with increasing **APyHC** concentration indicates that a higher energy barrier must be overcome for the EP mechanism to proceed^34^. All *E*_a_ values are significantly below the 80 kJ mol⁻¹ threshold; the adsorption mechanism is primarily physical in nature^35^. The enthalpy (ΔH ^≠^) and entropy (ΔS ^≠^) of activation for the metal dissolution reaction were computed using the transition state Eq^36^.:

4 $$\ln {\text{ }}(\:\frac{{\:I_{{\lim }} }}{T}) = \ln {\text{ }}(~\:\frac{R}{{Nh}}) + \:\frac{{\Delta \: \:S^{\neq}}}{R}~ - ~\:\:\frac{{\Delta \:H^{\neq}}}{{RT}}$$

where *N* is Avogadro’s number, *h* is Planck’s constant, and *T* is the temperature. The activation enthalpy (ΔH ^≠^) and entropy (ΔS ^≠^) for **APyHC** inhibitors were calculated from transition state theory plots of ln(Iₗ_i_ₘ/T) versus 1000/T (Fig. 4). The values of ΔH ^≠^ and ΔS ^≠^ were obtained from the slope (−ΔH ^≠^/R) and intercept (ln(R/Nh) + (ΔS ^≠^/R) of the linear regression, respectively, and are presented in Table 3. The increased positive enthalpy of activation (ΔH^≠^) confirms that the inhibitor elevates the energy barrier for CSs dissolution, effectively hindering the rate-determining step of the EP process. Also, the positive values of ΔH^≠^ for both uninhibited and inhibited systems confirm the endothermic nature of the CSs dissolution process, with values below 80 kJ/mol^37,38^. This is consistent with a physical adsorption mechanism, as values below this threshold typically indicate physisorption, while those around 100 kJ/mol suggest chemisorption. As the concentration of **APyHC** increases, a marked rise in the activation enthalpy (ΔH^≠^) is observed. This behavior indicates that the presence of the ionic liquid raises the energy barrier for anodic dissolution of carbon steel, thereby hindering the rate-determining step of the electropolishing process. The inhibitor molecules adsorb onto active surface sites, forming a physical barrier that limits metal dissolution and requires higher energy for the electrochemical reaction to proceed.

**Table 3:** Kinetic activation parameters CSs EP in test acidic solution.

ILs	C x10^5^ M	E_a_ (kJ/mol)	∆S^≠^ (J/mol.K)	∆H^≠^(kJ/mol)	∆G^≠^(kJ/mol)
*p*-APyHC	Blank	11.71	−158.37	9.21	56.80
	0.49	28.94	−105.85	26.45	58.25
	0.99	23.72	−124.49	21.22	58.63
	1.98	31.03	−101.82	28.53	59.13
	3.95	37.17	−82.38	34.67	59.43
	5.93	46.87	−50.98	44.37	59.69
*o*-APyHC	Blank	11.710	−158.37	9.21	56.80
	0.49	27.17	−111.05	24.67	58.05
	0.99	21.58	−130.92	19.08	58.42
	1.98	30.54	−102.62	28.04	58.88
	3.95	30.32	−104.35	27.82	59.18
	5.93	37.14	−82.67	34.65	59.49

The negative values of the activation entropy (ΔS^≠^), and consequently of TΔS^≠^, indicate a decrease in disorder during formation of the activated complex. This reflects an associative mechanism in which the activated state is more ordered than the reactants, consistent with the formation of an adsorbed inhibitor layer on the steel surface. The reduction in entropy is therefore attributed to the organization of **APyHC** molecules at the metal–solution interface, resulting in a compact, structured protective film^39^.

The interrelation between the activation enthalpy (ΔH^≠^), entropy (ΔS^≠^), and Gibbs free energy (ΔG^≠^) provides deep insight into the inhibition mechanism of **APyHC** during the electropolishing of carbon steel. These parameters are related by the transition-state Eq^40^:

5 $$\Delta G^{ \ne } = \Delta H^{ \ne } - {\text{ }}T{\text{ }}\Delta S^{ \ne }$$

The activation Gibbs free energy (ΔG^≠^) remains positive and increases slightly with inhibitor concentration, demonstrating that the dissolution process becomes thermodynamically less favorable in the presence of **APyHC**. This confirms the protective role of the ionic liquid film, which suppresses corrosion by increasing the energetic cost of metal dissolution.

Together, the increase in ΔH^≠^, the positive and increasing ΔG^≠^, and the negative ΔS^≠^ confirm that **APyHC** inhibits electropolishing primarily through a physisorption-controlled mechanism, in which an ordered ionic-liquid film forms on the carbon steel surface, raising the energy barrier for anodic dissolution and effectively suppressing corrosion^41^.

### Modeling of adsorption data

Adsorption isotherms detail the inhibitor’s behavior on metal surfaces, influenced by the electrolyte, inhibitor chemistry, and the metal’s properties during electropolishing. Adsorption is favored when the inhibitor-metal interaction energy exceeds that of water-metal. In this study, surface coverage (θ) values obtained from Galvanostatic measurements were quantified as a function of inhibitor concentration (*C*), revealing competitive or synergistic interactions at the interface and identifying the most suitable adsorption isotherm model^42^. The adsorption mechanism of *p-APyHC* and *o-APyHC* on CSs was elucidated by applying several adsorption isotherm models (Langmuir, Temkin, Frumkin, and El-Awady).

The Langmuir isotherm posits a monolayer of inhibitor molecules uniformly adsorbing onto a homogeneous metal surface without intermolecular interactions, with one molecule per site^32^. Conversely, the Temkin model incorporates lateral interactions between adsorbates under assumptions of uniform binding energy, a linear decline in adsorption heat with coverage, and notable interfacial effects^43^. The Frumkin isotherm accounts for both adsorption energy and adsorbate-adsorbate interactions, making it applicable to systems with significant intermolecular forces, such as corrosion inhibition and catalysis^44^. The El-Awady kinetic-thermodynamic isotherm can be employed to assess the number of water molecules displaced by one inhibitor molecule (*y*)on a metal surface. The inhibitor adsorbs onto the surface only if its binding affinity exceeds that of water; otherwise, adsorption does not occur.

**Fig. 5:**
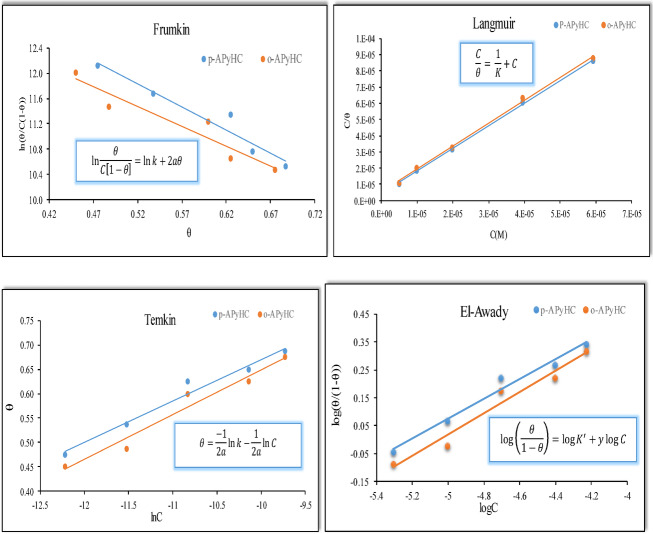
The series of adsorption isotherms investigated.

**Fig. 6:**
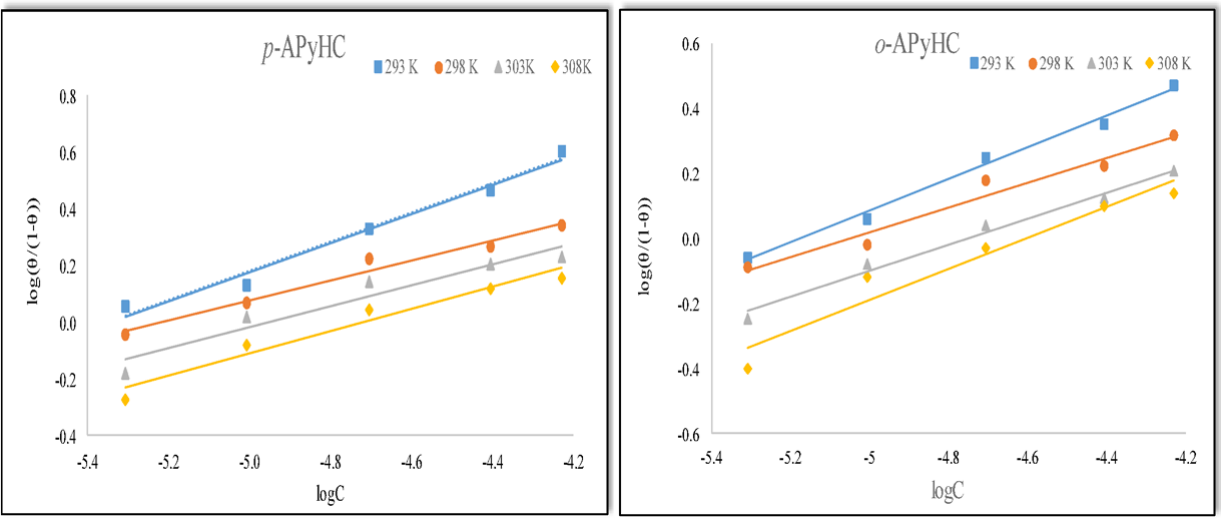
El-Awady adsorption isotherm of **APyHC** on CSs surface in 8 M H_3_PO_4_ at different studied temperatures.

Applying these models clarified the nature of **APyHC** adsorption onto the CSs’surface, providing insight into its effectiveness as a dissolution inhibitor, as summarized in Figs. 5 and 6, and Table 4. Ultimately, the El-Awady model demonstrated the best fit to experimental data, indicating its greater accuracy in characterizing the adsorption mechanism^45^. The adsorptive equilibrium constant K_ads_ is given by: *K*_*ads*_ *= K* ^,^^*(1/y)*^. The Parameter *1/y* provides critical insight into the adsorption behavior of the inhibitor. Values below unity (*1/y < 1*) indicate multilayer adsorption on the metal surface, whereas values above unity (*1/y > 1*) suggest that individual inhibitor molecules interact with multiple active sites^43^. The adsorption equilibrium constant (*K*_*ads*_) serves as a quantitative measure of the interaction affinity between inhibitor molecules and the metal surface within the electrolyte (Table 5).

Higher *K*_*ads*_ values indicate thermodynamically favorable adsorption, promoting greater surface coverage and, consequently, improved corrosion inhibition efficiency^42^. The inverse correlation between *K*_*ads*_ and temperature suggests that higher thermal energy disrupts the adsorption equilibrium, reducing inhibition efficiency. This behavior indicates that the adsorption of the investigated inhibitors onto CSs becomes less favorable at higher temperatures. Notably, *p-APyHC* exhibits greater *K*_*ad*s_ values than *o-APyHC* at identical temperatures, reflecting its stronger adsorption affinity for the CSs surface. Accordingly, the inhibitor’s strong adsorption affinity allows it to form a cohesive protective layer on the surface, which acts as a barrier against corrosive agents and consequently slows the EP process^45^.

The Gibbs free energy of adsorption (ΔG^o^_ads_), a fundamental thermodynamic parameter, was calculated using the Eq. (6)^46^:

6 $$\Delta G^{o}_{ads}{\text{ }} = {\text{ }} - RT{\text{ }}\ln {\text{ }}(55.5{\text{ }}K_{ads})$$

where *R*, *T*, and *55.5 M* are the universal gas constant, thermodynamic temperature (293–308 K), and the molar concentration of water in the solution. The ΔG^o^_ads_ serves as a crucial indicator for distinguishing between different adsorption mechanisms. Physisorption, dominated by electrostatic interactions between charged inhibitor molecules and the metal surface, is typically associated with ΔG^o^_ads_ values less negative than − 20 kJ·mol^− 1^. In contrast, chemisorption involving the sharing or transfer of electron pairs (or π-electrons) from organic inhibitors to the metal surface to form coordinate covalent bonds is characterized by ΔG^o^_ads_ values more negative than − 40 kJ·mol^− 1^^47^ Values in the intermediate range (−40 to −20 kJ·mol⁻¹) suggest a mixed adsorption mechanism combining both physical and chemical adsorption^48,49^. The calculated ∆G^o^_ads_ values for both *p-*APyHC and *o-*APyHC at each experimental temperature are summarized in Table 5. The ∆G^o^_ads_ values for p-APyHC and o-APyHC range from − 39.77 to −38.07 kJ·mol⁻¹ and − 38.78 to −37.40 kJ·mol⁻¹, respectively, within the temperature range of 293–308 K, confirming a spontaneous adsorption process governed by a combination of chemisorption and physisorption mechanisms (physicochemical). The highly negative ∆G^o^_ads_ values elucidate the spontaneous adsorption of inhibitor molecules and the stability of the adsorbed layer^42^. The adsorption enthalpy (ΔH^o^_ads_) was determined using the Van’t Hoff equation (Eq. 7)^50^:

7 $$\ln {\text{ }}K_{ads}{\text{ }} = \frac{{ - \Delta H_{ads}}}{{RT}}{\text{ }} + {\text{ }}Constant$$

**Table 4:** Thermodynamic parameters derived from **APyHC** adsorption isotherm data.

IL	Isotherms	R^2^	K_ads._	∆G^o^_ads._kJ/mol	Parameters	
*p*-APyHC	Langmiur	0.9992	-	-	Slope	1.3981
*o*-APyHC		0.9979	-	-		1.4129
*p*-APyHC	Frumkin	0.9399	6.1073E+06	−48.66	a	−3.65
*o*-APyHC		0.9047	2.5307E+06	−46.48	a	−3.15
*p*-APyHC	Temkin	0.9734	5.9545E+07	−54.31	a	−5.89
*o*-APyHC		0.9623	2.4871E+07	−52.14	a	−5.41
*p*-APyHC	El Awady	0.9769	1.6292E+05	−39.68	y	0.35
					K'	70.24
*o*-APyHC		0.9637	1.1162E+05	−38.75	y	0.38
					K'	83.40

**Fig. 7:**
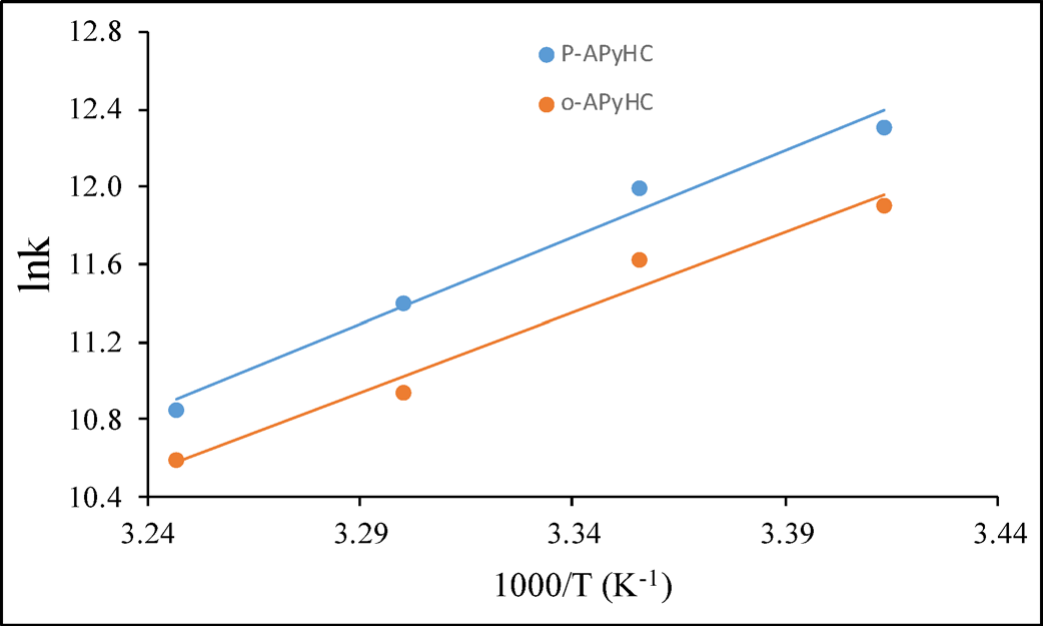
Van Hoff equation for **APyHC** adsorption in 8M H_3_PO_4_.

A linear correlation was observed when plotting *ln K*_*ads*_ versus *1/T*, as illustrated in Fig. 7**.** The slope of this linear relationship corresponds to *− ΔH*^*o*^_*ads*_*/R*, enabling determination of the enthalpy of adsorption. The negative ΔH^o^_ads_ values confirmed the exothermic nature of the inhibitor’s adsorption process on mild steel. These results corroborate the observed decrease in inhibition efficiency with rising temperature. The standard entropy of adsorption (∆S^°^_ads_) can be determined using Eq. (8)^51^:

8 $$\Delta G^{ \circ } _{{ads}} = \Delta H^{ \circ } _{{ads}} - T\Delta S^{ \circ } _{{ads}}$$

**Table 5:** El-Awady isotherm fitting results.

T(K)	y	K'	K_ads_	∆G^o^_ads_(kJ/mol)	R^2^	∆H^o^_ads_(kJ/mol)	∆S^o^_ads_(J/mol.K)
IL	*p*-APyHC						
293	0.5137	557.70	221892.30	−39.77	0.9780	−74.59	−119.00
298	0.3543	70.24	162594.26	−39.68	0.9700		
303	0.3718	69.29	89285.31	−38.84	0.9240		
308	0.3903	69.04	51524.81	−38.07	0.9541		
IL	*o*-APyHC						
293	0.488	333.12	147659.81	−38.78	0.9915	−69.38	−104.10
298	0.3806	83.40	111624.85	−38.74	0.9637		
303	0.4037	82.58	56018.05	−37.66	0.9850		
308	0.4767	155.78	39749.23	−37.40	0.9373		

**Fig. 8:**
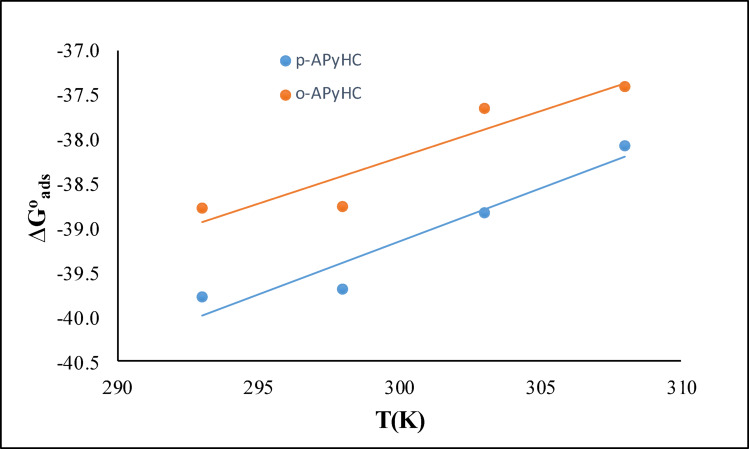
ΔG^°^_ads_ as a Function of Temperature for variable concentrations of **APyHC**.

As obtained slope from Fig. 8, the negative ΔS^o^_ads_ values reflect a decrease in system disorder during adsorption, suggesting the formation of ordered inhibitor-surface complexes. While dissolution typically increases entropy, this observation can be explained by the replacement of disordered water molecules from the metal interface with more structured ionic liquid adsorbates^52^.

### Integrated experimental and computational surface analysis

#### SEM/EDX

**Fig. 9:**
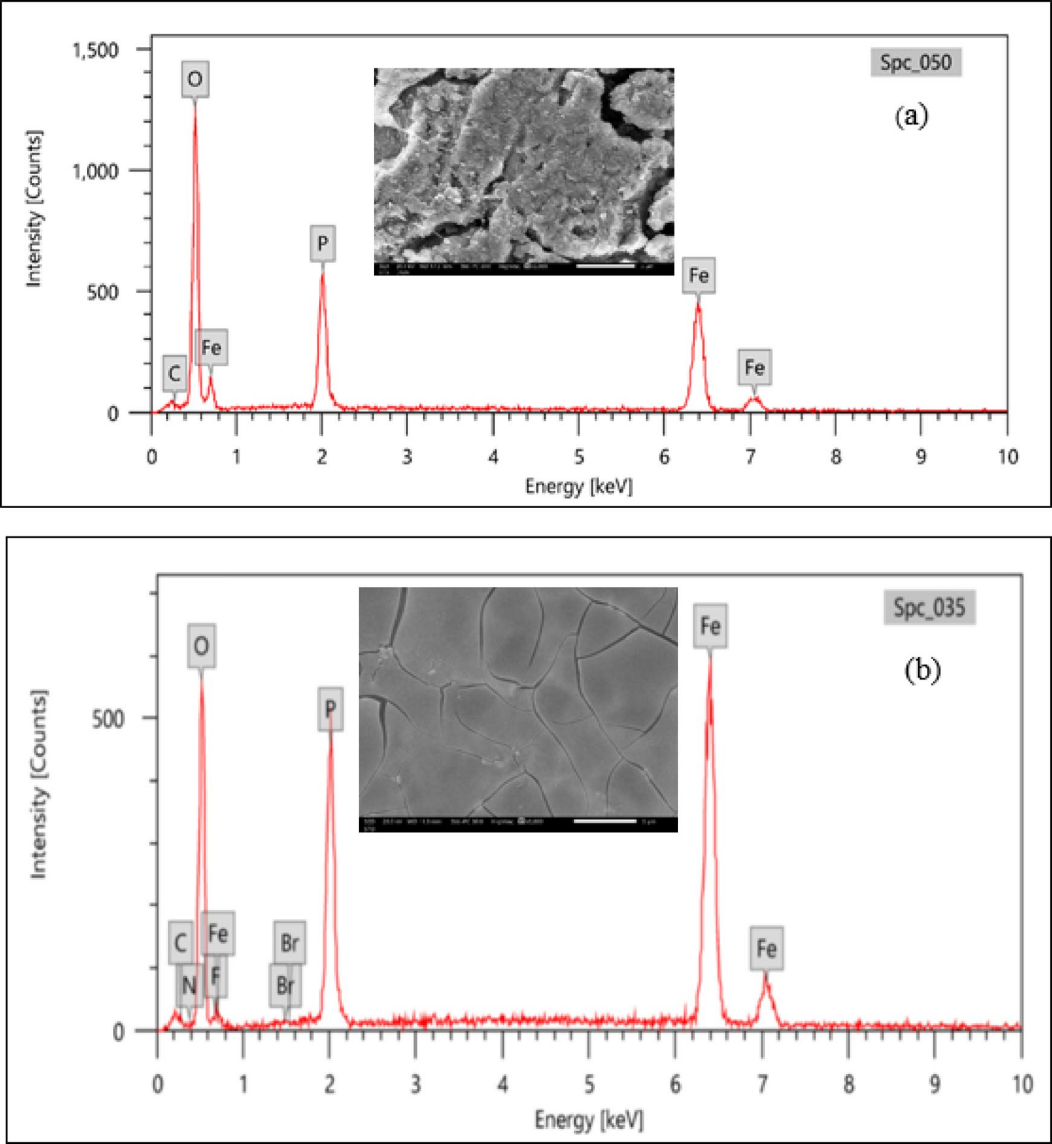

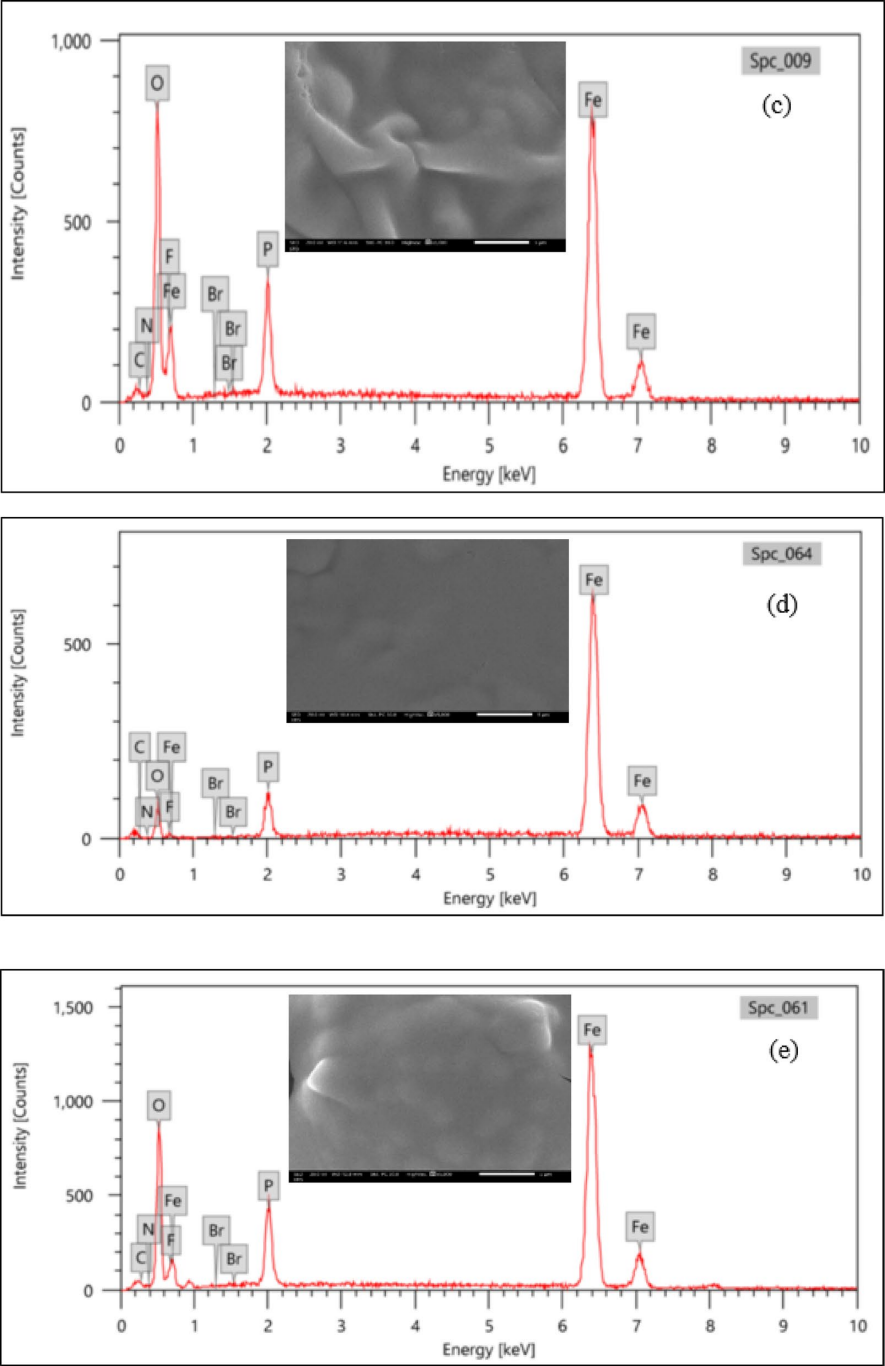
SEM images and EDX spectra of a) Blank, b) *p*-APyHC (4.94× 10^–6^M) (293 K), c) *p*-APyHC (5.928× 10^–5^ M) (308 K), d) *p*-APyHC (5.928× 10^–5^ M) (293 K), and e) *p*-APyHC (5.928× 10^–5^ M) (293 K).

Scanning Electron Microscopy (SEM) and Energy-Dispersive X-ray Spectroscopy (EDX) were used in tandem to provide complementary insights into surface morphology and elemental composition during the EP process^34^. Figure 9 shows the EP SEM image of the uninhibited CSs exposed to 8 M H₃PO₄, which reveals significant surface deterioration. This severe corrosion is driven by the aggressive action of phosphate ions (PO₄³⁻), which facilitate metal dissolution and the buildup of corrosion products. The absence of an inhibitor leaves the surface vulnerable to accelerated damage and material loss.

Conversely, the addition of varying concentrations of **APyHC** at different temperatures significantly improved the condition of the carbon steel (CS). The SEM image of the sample treated with *p*-**APyHC** (Fig. 9) reveals a uniform, planar surface devoid of cracks and pits, indicating superior corrosion mitigation compared to *o-APyHC* (Fig.9). This enhanced surface integrity, attributable to the formation of a protective film, effectively blocks phosphate-ion infiltration and substantially reduces corrosive interactions with the acidic medium, resulting in smoother surfaces with minimal defects^53^.

Energy-Dispersive X-ray (EDX) spectroscopy (Fig. 9; Table 6) corroborates the SEM findings. Treatment with **APyHC** led to a significant restoration of iron content, increasing from 37.32% in the corroded state to 56.15%, 62.50%, 69.85%, and 80.00%. Concurrently, the substantially reduced oxygen and phosphorus content indicates a marked decrease in corrosion products, underscoring the effectiveness of these ILs in preventing oxidation and phosphate adsorption onto the carbon steel^54^. The detection of nitrogen and bromine confirms the adhesion of **APyHC** to the surface. The observed carbon signals are attributed to the organic components of the adsorbed ionic liquids.

Collectively, SEM and EDX provide a comprehensive assessment of structural and compositional changes, confirming the formation of a protective layer and reduced corrosive attack. This combined analysis validates the trends from gravimetric and electrochemical studies, offering a holistic view of the inhibition mechanism at microstructural and chemical levels.

**Table 6:** Comparative EDX Analysis of steel surface Composition in 8 M H₃PO₄ with and without **APyHC.**

Mass %	Fe	C	O	P	N	Br	%IE
a)Blank	37.32±0.02	1.21±0.01	54.81±0.93	15.66±0.11	nd	nd	0.00
B) *p*-APyHC (4.94 × −10⁻^6^ M) (293 K)	56.16±0.82	1.49±0.07	27.49±0.42	2.711±0.33	0.21±0.88	0.18±0.14	53.33
c) *p*-APyHC (5.928 × −10⁻⁵ M) (308 K)	62.50±0.76	2.37±0.11	26.09±0.36	8.60±0.22	0.23±0.08	0.21±0.11	58.95
d) *p*-APyHC (5.928 × −10⁻⁵ M) (293 K)	88.00±1.21	4.85±0.21	4.32±0.21	2.03±0.25	0.31±0.07	0.29±0.13	80.00
e) *o*-APyHC (5.928 × −10⁻⁵ M) (293 K)	69.85±0.67	3.49±0.07	18.62±0.26	7.48±0.18	0.29±0.06	0.27±0.11	74.67

#### AFM-based roughness analysis under harsh conditions

**Fig. 10:**
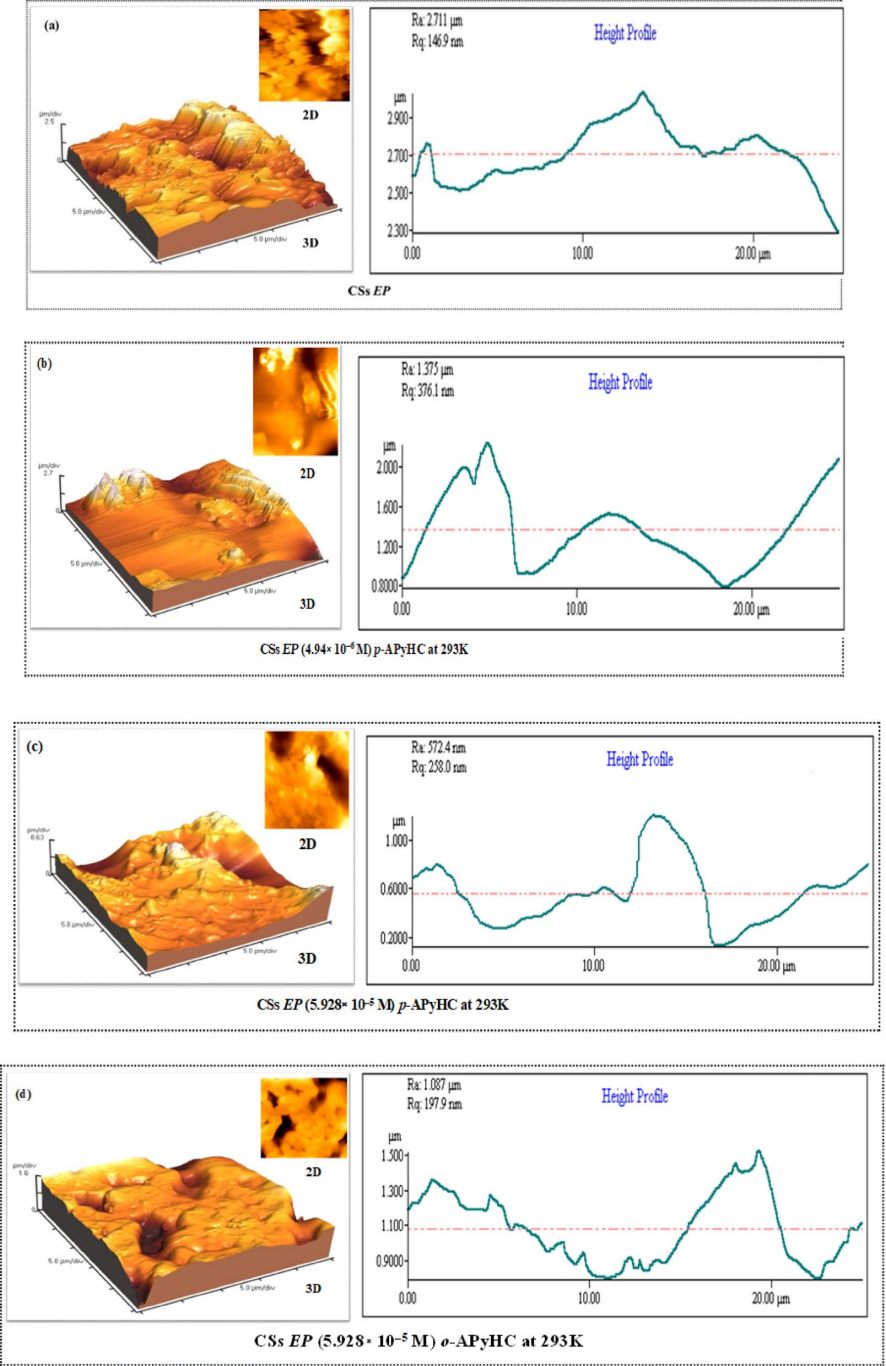

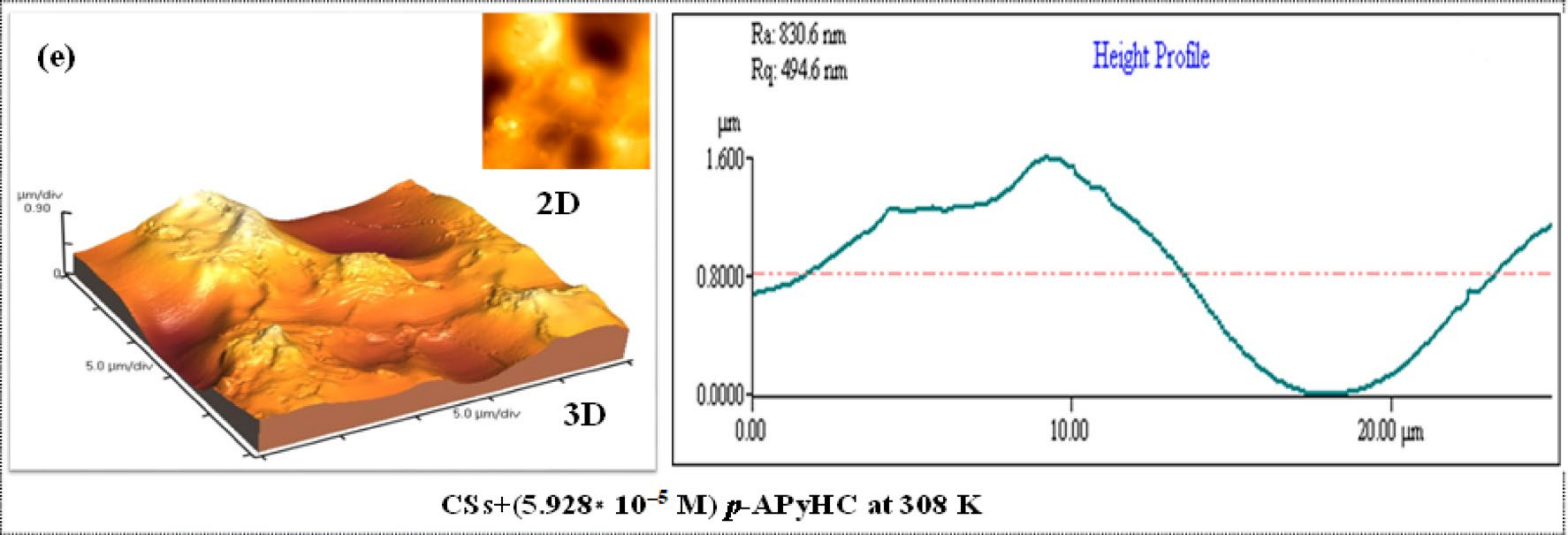
Topography evolution of the CSs’ surface during AFM measurements.

In situ AFM was used as an essential tool to quantitatively measure micro- and nanoscale surface changes on CSs during EP experiments in 8 M H₃PO₄, with and without specific inhibitors **APyHC**. The 3D AFM data, which revealed that temperature and inhibitor concentration reduce surface roughness (Fig. 10), provided quantitative support for the qualitative morphological and elemental profiles obtained from SEM/EDX^34^.

Quantitative analysis of surface roughness parameters, including the average roughness (Ra), root-mean-square roughness (Rq), and peak-to-valley depth (Rp-v), served as definitive metrics for evaluating the protective efficacy of the inhibitors^55^. Exposure to the uninhibited acid solution resulted in severe corrosion damage, producing a highly irregular surface with prominent bumps and superficial pits. This severe degradation was quantified by a significantly elevated average roughness of 2.711 ± 0.21 μm, indicative of vigorous anodic dissolution and confirming the solution’s inherent aggressiveness (Fig. 10a).

In stark contrast, the addition of the inhibitors *p*-**APyHC** and *o*-**APyHC** at their optimal concentrations (5.928 × 10⁻⁵ M, 293 K) yielded a dramatic improvement in surface preservation. *P*-**APyHC** proved to be the most effective inhibitor, generating the lowest average roughness value of approximately 0.572 ± 0.02 μm. *o*-**APyHC** also produced a substantial protective enhancement. The corresponding AFM topographies (Fig. 10) visually corroborate these findings, showing remarkably smooth, uniform surfaces. The pronounced reduction in roughness parameters, especially the maximum peak height of the CSs’ surface, reaches approximately 2.87 ± 0.11 μm in the blank solution (Fig. 10a). In addition to *p*-**APyHC**, the maximum peak height is significantly reduced to 630 nm at 5.928 × 10⁻⁵ M, 293 K (Fig. 10c). This provides direct evidence of a coherent protective film formation. These films act as effective barriers, significantly limiting direct interaction between the metal surface and corrosive species and thereby preventing CSs’ deterioration.

As we concluded from the last data, CSs’ samples after electropolishing in H_3_PO_4_ become microsmooth while retaining nanoroughness, which can be beneficial for many applications.

#### XPS surface analysis

**Fig. 11:**
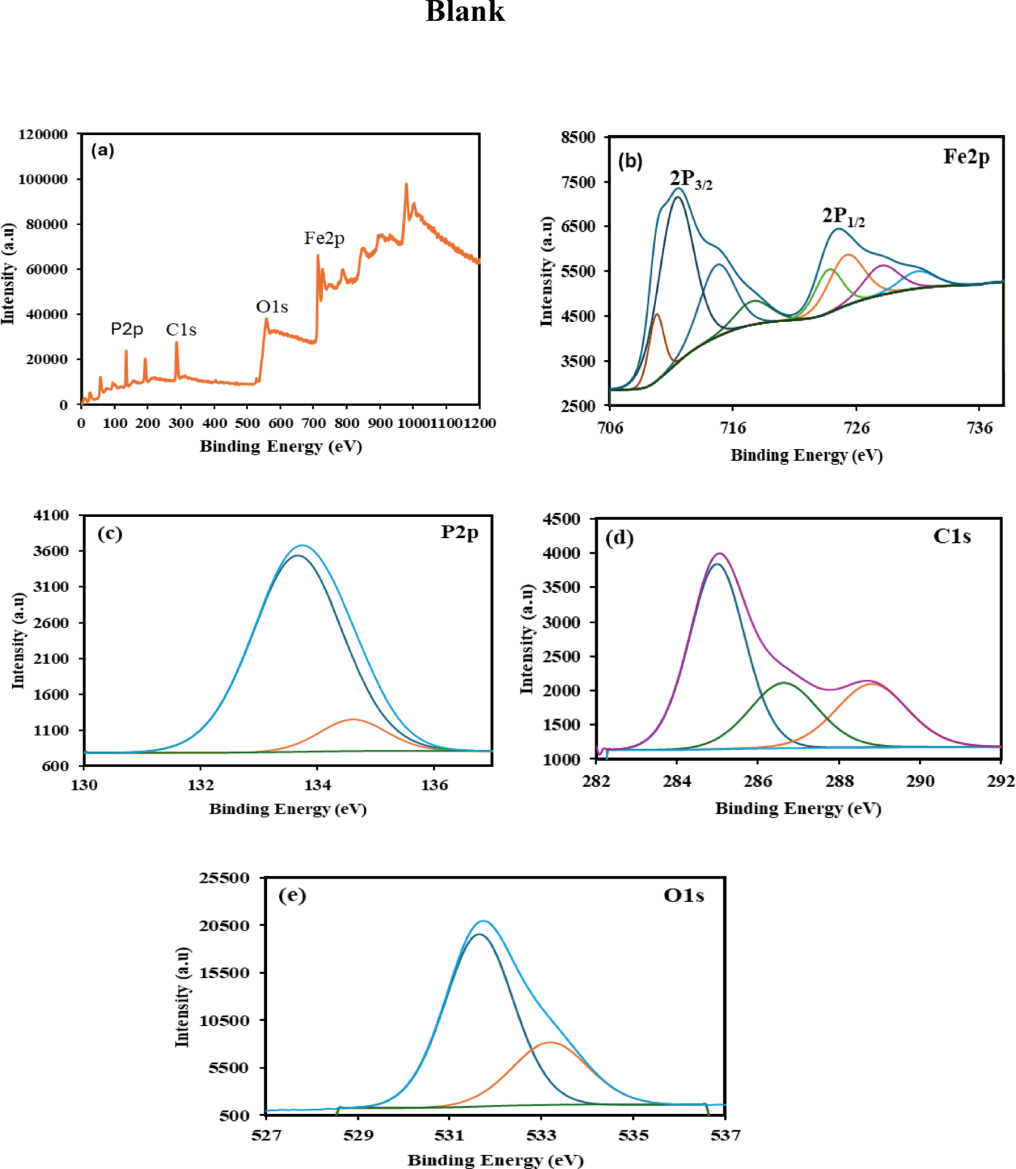
XPS analysis for CSs surface (blank) exposed to 8 M H_3_PO_4_: (**a**) XPS survey scan and XPS spectrum of (**b**) Fe 2p, (**c**) P 2p, (**d**) C1s and (**e**) O1s.

**Fig. 12:**
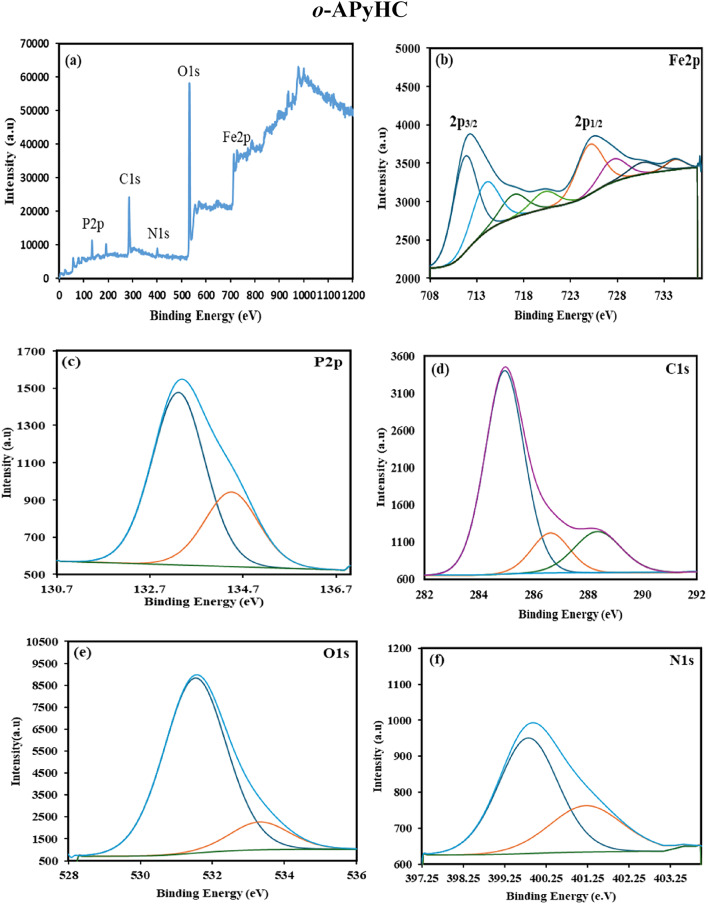
(**a**) XPS survey scan and XPS spectrum of (**b**) Fe 2p, (**c**) P2p, (**d**) C1s, (**e**) O1s and (**f**) N1s for *o-APyHC* inhibitor.

**Fig. 13:**
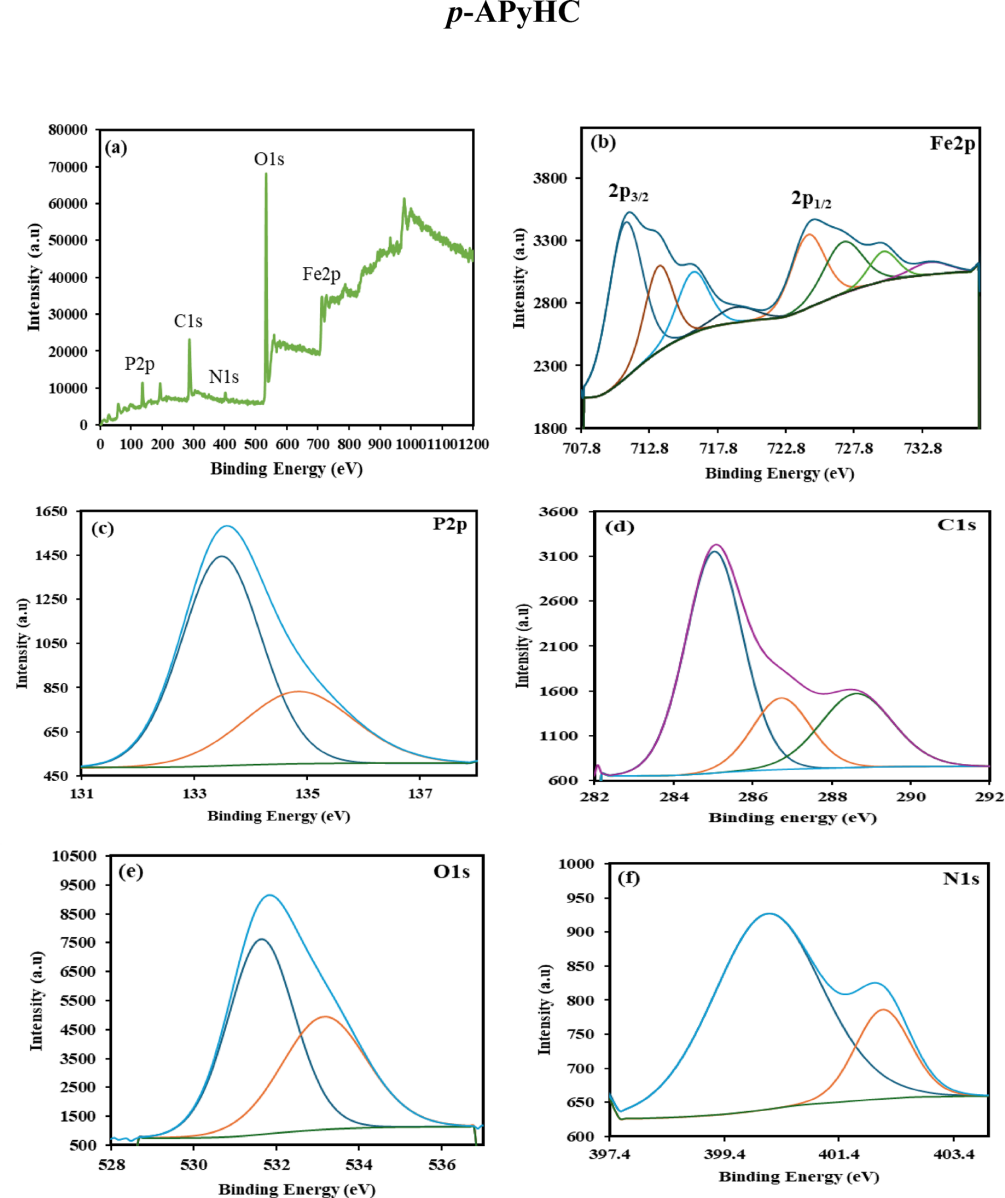
(**a**)XPS survey scan and XPS spectrum of(**b**)Fe 2p, (**c**) P 2p, (**d**)C1s, (**e**) O1s and(**f**)N1s for *p*-APyHC inhibitor.

XPS analysis was employed to characterize the adsorption of **APyHC** inhibitors on CSs, with a specific focus on elucidating the chemical composition and binding configuration of the resulting protective film. The technique is uniquely capable of probing the specific bonding interactions between heteroatoms in the inhibitor molecules and the steel substrate. A comparative analysis of the surface before and after IL adsorption was pivotal for uncovering the underlying inhibition mechanism. Critically, the XPS survey spectra in (Figs. 11a and 12a, and 13a) provide definitive evidence of successful adsorption, marked by the emergence of a nitrogen (*N*) peak exclusively on the treated surface, thereby confirming the incorporation of inhibitor molecules onto the steel substrate.

The initial characterization of the phosphoric acid-treated CSs surface (the “blank,” Fig. 11) established a critical baseline of its chemical state before inhibitor introduction. This analysis confirmed the presence of several iron oxides, identified as *Fe₃O₄* or *FeO* (associated with *Fe*^*+*^*²*) and *Fe₂O₃* (associated with *Fe*^*+*^*³*). These species were characterized by two primary *Fe 2p* peaks at binding energies of 711.48 eV (2p₃/₂) and 724.48 eV (2p₁/₂). Further examination revealed additional chemical features: the P 2p spectrum exhibited two deconvoluted peaks at 133.69 eV and 134.66 eV, consistent with *P-O* and *PO*_*4*_^*−3*^ species, respectively. The *C 1s* spectrum was resolved into three distinct components at 284.99 eV, 286.64 eV, and 288.81 eV, corresponding to *C–H/C–C*,* C–N*, and *C = O* bonds, respectively. Complementing this, the O 1 s spectrum showed two peaks at 531.64 eV and 533.18 eV, attributed to *Fe–O/C = O* and *O–C* bonds, respectively. This comprehensive profiling of the blank surface provided a definitive reference for evaluating subsequent chemical changes.

Following inhibitor adsorption, the emergence of new *N 1s* peaks served as direct evidence of a chemical interaction between the inhibitor and the substrate. As presented in (Fig. 13), the spectrum for the *p*-**APyHC** inhibitor reveals two distinct *N 1s* peaks at 400.15 eV and 402.33 eV, assigned to *C-N/N-N* and *Fe-N/N*^+^ species, respectively. This provides conclusive evidence for the formation of a coordinated protective layer. This interaction is further substantiated by systematic shifts in the binding energies of the core *Fe*, *P*, and *O* peaks post-adsorption, confirming their active participation in forming a stable surface coating. Moreover, this inhibition mechanism is consistent across different inhibitors, as a nearly identical *N 1s* spectral profile is observed for *o-APyHC* (Fig. 12), with all pertinent peak shift data detailed in Table 7.

These XPS findings provide molecular-level confirmation of the adsorption and inhibition mechanisms inferred from electrochemical and microscopic analyses.

**Table 7:** XPS-derived binding energies for **APyHC** inhibited samples.

*Element*	Binding energy	Assignment	Ref.	*Element*	Binding energy	Assignment	Ref.
*o*-APyHC + Fe				*p*-APyHC + Fe			
C	284.96	C-C/C = C/C-H	^56^	C	285.03	C-N/C-H	^57^
	286.54	C-N	^56,57^		286.72	C-N	^56,57^
	288.06	C = O	^58^		288.62	C = O	^57^
N	400.06	N-C/N-N	^59^	N	400.15	N-C/N-N	^58^
	402.33	N-Fe/N⁺	^60^		402.16	N-Fe/N⁺	^60,61^
O	531.52	Fe-O/C = O	^62^	O	531.55	Fe-O/C = O	^62^
	533.31	Fe_2_O_3_	^63^		532.75	C-O	^64^
P	133.51	P-O	^64^	P	133.48	P-O	^64^
	134.71	PO4^− 3^	^65^		134.24	PO4^− 3^	^65^
Fe	713.92	Fe2p_3/2_	^66^	Fe	712.09	Fe2p_3/2_	^67^
	725.09	Fe 2p_1/2_	^66^		723.74	Fe 2p_1/2_	^67^

#### Computational insights into the EP mechanism

**Fig. 14:**
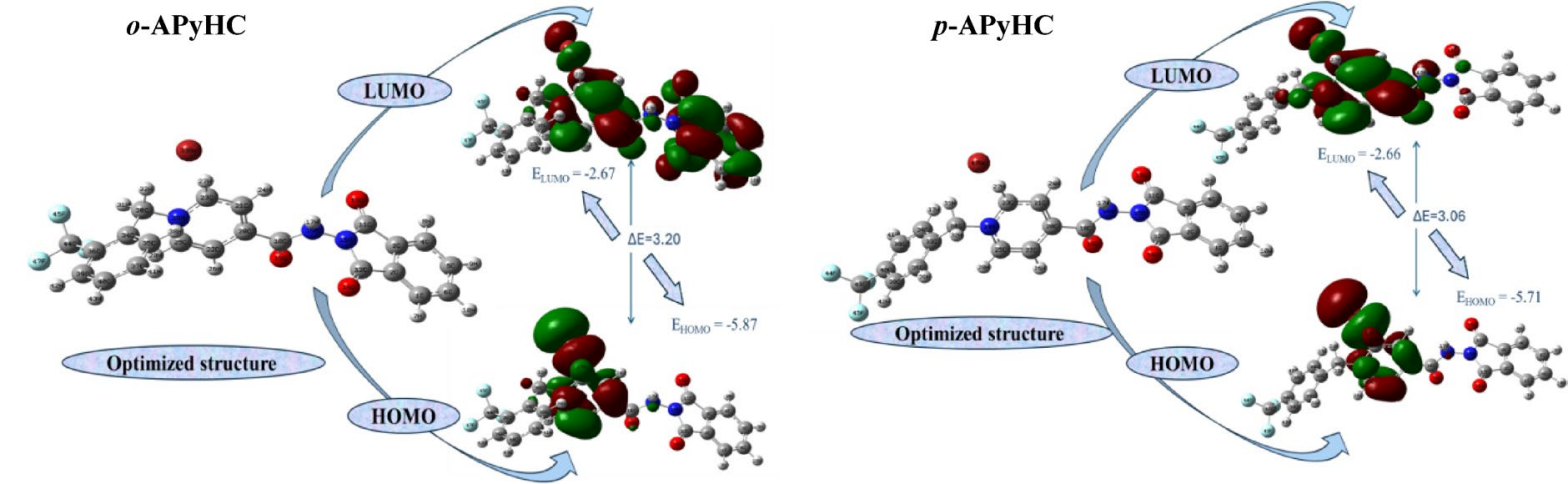
HOMO and LUMO orbitals for **APyHC**.

Alongside the experimental investigations, density functional theory (DFT) calculations were conducted to gain deeper insights into the molecular-level mechanism of corrosion inhibition. The molecular geometries of the two ionic liquids were optimized using the B3LYP functional with the 6-311G(d, p) basis set implemented in Gaussian 09, and the optimized structures were further visualized using Gauss View 5.0 (Fig. 14). Subsequently, a comprehensive set of quantum chemical descriptors was evaluated, including the frontier orbital energies (E_HOMO_ and E_LUMO_), energy gap (ΔE), electron transfer fraction (ΔN), electronegativity (χ), global hardness (η), softness (σ), chemical potential (µ), ionization potential (I), electron affinity (A), dipole moment (µd), electrophilicity (ω), and nucleophilicity in both gas and aqueous phases, as summarized in Table 8^68^. Since the corrosion process occurs in an aqueous acidic medium, solvent effects must be included to obtain a more realistic representation of the inhibitor–metal interactions under practical conditions. Therefore, aqueous-phase calculations were performed using the polarizable continuum model (PCM), enabling a better evaluation of the electronic behavior of the inhibitor molecules in solution. The obtained quantum descriptors in the aqueous phase exhibited trends comparable to those observed in the gas phase, confirming the consistency and reliability of the theoretical calculations. However, slight variations in the electronic parameters were observed due to solvent-induced polarization effects, which significantly influence charge distribution, molecular polarity, and electron transfer capability.

**Fig. 15:**
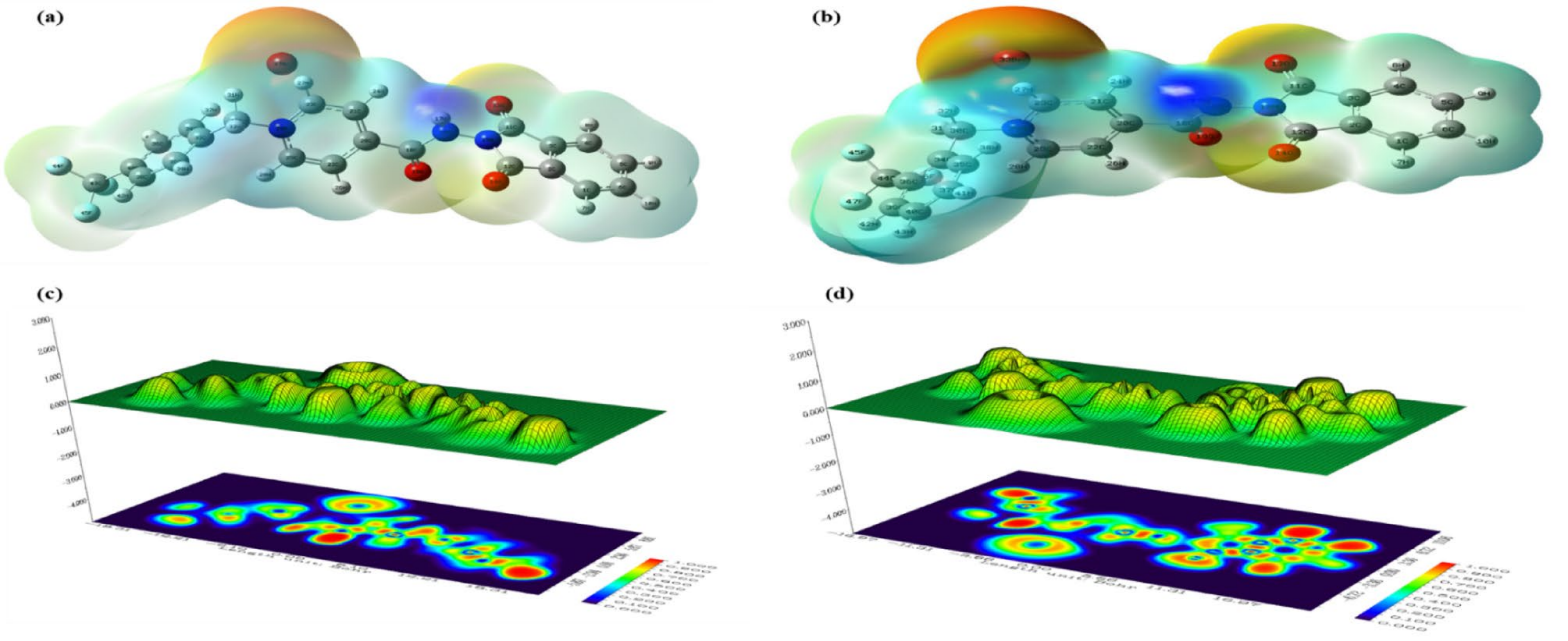
MEP map of (**a**) *p-APyHC*, (**b**) *o*-**APyHC,** ELF color-filled maps in XY-plane, and isosurfaces of valence electrons overview (**c**) *p*-**APyHC**, (**d**) *o*-**APyHC**.

Frontier molecular orbital (FMO) analysis provided valuable insights into the electronic interactions between the inhibitor molecules and the steel surface. A higher E_HOMO_ value reflects a stronger electron-donating capacity, enhancing the likelihood of charge transfer to the vacant d-orbitals of *Fe* atoms. In contrast, a lower E_LUMO_ value indicates a greater propensity to accept back-donation from filled metal orbitals, thereby stabilizing donor–acceptor interactions^69^. Comparative analysis revealed that ***p-APyHC*** exhibits both a significantly elevated E_HOMO_ and a reduced E_LUMO_ relative to ***o-APyHC*** in both gas and aqueous phases (Table 8), highlighting its superior dual electron donor–acceptor character. This favorable electronic configuration rationalizes the experimentally observed enhancement in ***p-APyHC’s*** inhibition performance. Furthermore, the presence of solvent molecules in the aqueous phase increased the polarity of the inhibitors and enhanced their interaction with the metal surface, thereby improving adsorption characteristics.

The energy gap (ΔE = E_LUMO_ – E_HOMO_), a fundamental indicator of chemical reactivity, showed an inverse relationship with inhibitor efficiency: smaller ΔE values promote stronger adsorption and enhanced interaction with the metal surface^70^. In this regard, ***p-APyHC*** demonstrated a notably reduced ΔE compared to ***o-APyHC*** in both phases, confirming its greater chemical reactivity and adsorption potential. The relatively lower ΔE values obtained in aqueous solution further suggest that the inhibitor molecules become more reactive in the presence of solvent effects, which facilitates adsorption onto the steel surface and improves corrosion protection efficiency. Such findings provide a quantum-level explanation for ***p-APyHC’s*** superior protective efficiency.

Additionally, the electron transfer fraction (ΔN) was employed to quantitatively assess the capacity of inhibitor molecules to donate electrons to the metallic surface. Positive ΔN values (ΔN > 0) indicate spontaneous electron transfer from the inhibitor to vacant metal orbitals, whereas negative values suggest the reverse^71^. Both inhibitors exhibited positive ΔN values in the gas and aqueous phases, consistent with their adsorption capability. Importantly, ***p-APyHC*** displayed a considerably higher ΔN value than ***o-APyHC***, underscoring its enhanced charge transfer ability. This stronger electron donation capacity further corroborates the experimentally observed superior inhibition efficiency of ***p-APyHC***.

The concepts of global hardness (η) and global softness (σ) are fundamental descriptors in conceptual DFT, widely employed to rationalize chemical reactivity trends^72^. According to Pearson’s hard and soft acids and bases (HSAB) principle, compounds with smaller HOMO–LUMO gaps exhibit higher polarizability, which in turn enhances their chemical reactivity while reducing kinetic stability, classifying them as soft species. Such softness promotes stronger interactions with metallic surfaces by facilitating charge redistribution. In the present study (Table 8), ***p***-**APyHC** exhibited a markedly higher softness (σ = 0.654 eV) compared with *O-APyHC* (σ = 0.624 eV), alongside a correspondingly lower hardness (η = 1.528 eV vs. 1.601 eV) in the gas phase. This electronic profile indicates that ***p***-**APyHC** is more chemically reactive and possesses a greater tendency to adsorb onto steel surfaces, thereby reinforcing its superior inhibition performance.

Electronegativity (χ) is another crucial quantum descriptor that reflects a molecule’s tendency to attract electrons. In corrosion inhibition studies, lower electronegativity values are generally favorable, as they imply a stronger ability of the inhibitor to donate electrons to the metal’s vacant d-orbitals, facilitating protective film formation through adsorption^73,74^. Computed values demonstrate that ***p-*APyHC** exhibits lower electronegativity than ***o-*APyHC** in both phases, consistent with its enhanced donor characteristics and improved inhibition efficiency. Moreover, the dipole moment (µd), which reflects molecular polarity, increased in the aqueous phase due to solvent effects. The higher dipole moment observed for ***p-*APyHC** indicates a stronger interaction with the metallic interface and enhanced adsorption capability in aqueous medium. These findings collectively demonstrate that including solvent effects improves agreement between theoretical calculations and experimental observations, confirming that **p-APyHC** exhibits superior corrosion inhibition performance owing to its favorable electronic structure and enhanced adsorption properties in both gas and aqueous phases.

- Electrostatic Potential Distribution and Reactive Centers.

Molecular electrostatic potential (MEP) analysis provides a robust computational framework for visualizing the spatial distribution of electron density and identifying the most chemically reactive regions within inhibitor molecules. In these maps, negative potential domains (red) correspond to nucleophilic sites with high electron-donating ability, while positive potential areas (blue) indicate electrophilic centers capable of accepting electrons. The electron density distribution is represented as a continuous gradient, decreasing from red (maximum density) through orange, yellow, and green to blue (minimum density), thereby providing a quantitative visualization of electronic polarization across the molecular structure^75^.

**Table 8:** The calculated quantum chemical indices in eV for the investigated **APyHC** at DFT/B3LYP/6-311G(d,p) in the gas and aqueous phases.

State	Gas		Aqueous	
Cpds	p-APyHC	o-APyHC	p-APyHC	o-APyHC
E_HOMO_ (eV)	−5.71	−5.87	−5.63	−5.70
E_LUMO_ (eV)	−2.66	−2.67	−1.86	−1.96
∆Egap (eV)	3.06	3.20	3.77	3.74
I	5.71	5.87	5.63	5.70
A	2.66	2.67	1.86	1.96
X	4.19	4.27	3.75	3.83
Pi	−4.19	−4.27	−3.75	−3.83
µd (Debye)	10.04	10.56	15.31	17.00
ƞ (eV)	1.53	1.60	1.88	1.87
σ (eV^−1^)	0.65	0.62	0.53	0.53
ω (eV)	5.73	5.69	3.73	3.92
∆Eb.d (eV)	−0.38	−0.40	−0.47	−0.47
∆N (e)	0.92	0.85	0.86	0.85
T.E (Hartee)	−4111.39	−4111.38	−4001.45	−4000.94
∆E_steel/inh_ (eV)	1.30	1.16	1.40	1.34
ω^-^ (eV)	8.01	8.02	5.84	6.07
ω^+^ (eV)	3.83	3.75	2.09	2.24
∆ω^±^ (eV)	3.70	3.63	1.92	2.07

**Fig. 16:**
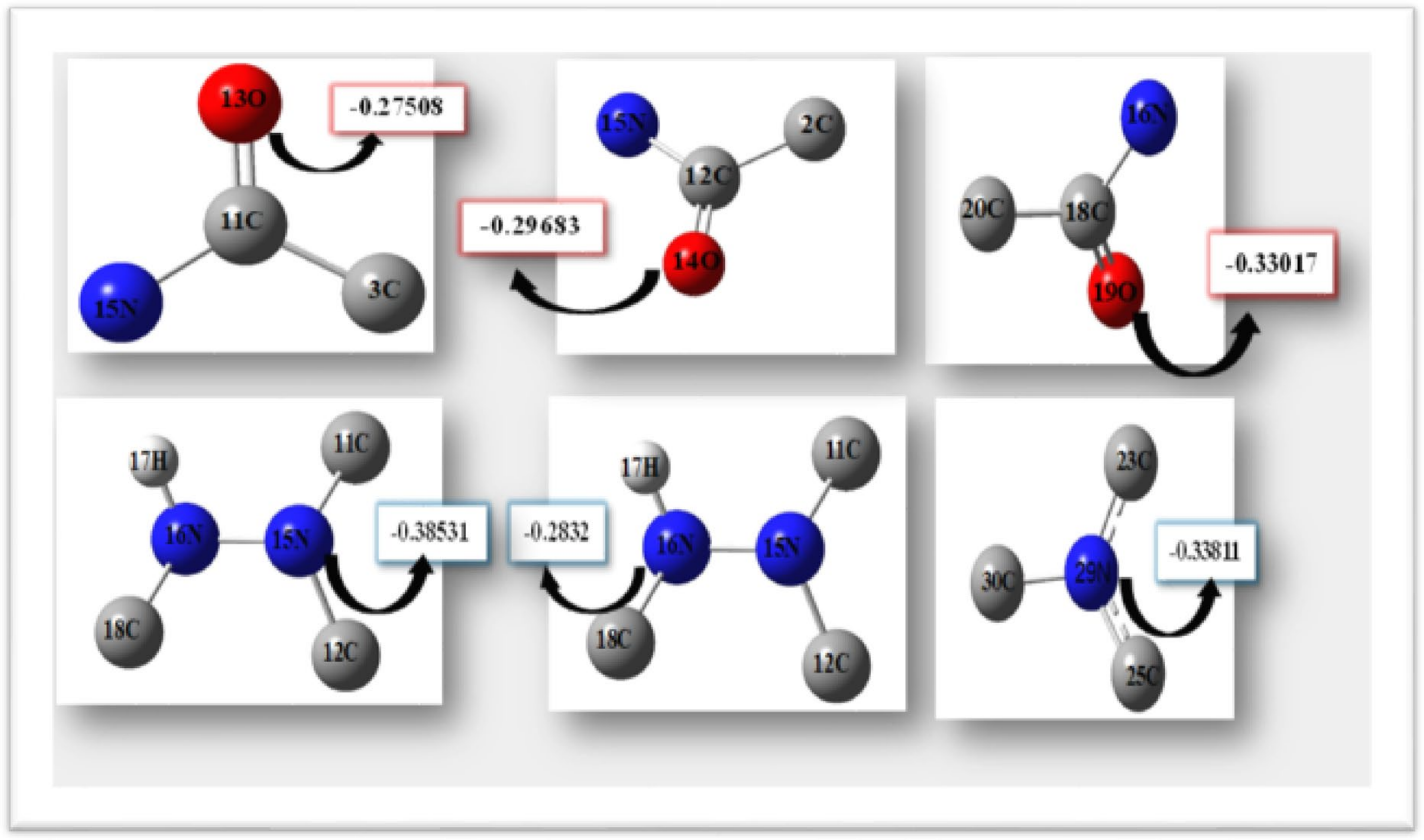
Mulliken charge distribution of the most negative centers in **APyHC**.

As illustrated in Fig. 15a and b, both ***p***-**APyHC** and ***o***-**APyHC** display distinct electron density localization, with the most pronounced negative potential regions (yellow to red) concentrated around oxygen and nitrogen atoms, highlighting them as the primary donor sites. In contrast, the lower-electron-density areas (green to blue) are associated with specific carbon atoms. To further validate these observations, a Mulliken population analysis was performed, which confirmed the presence of multiple active centers, including O13, O14, N15, N16, O19, and N29, in both molecules, as shown in Fig. 16. The presence of multiple electron-rich centers is particularly advantageous for corrosion inhibition, as atoms bearing higher negative partial charges serve as effective donor sites for electron transfer to the vacant d-orbitals of iron. This donor–acceptor interaction enhances the adsorption of inhibitor molecules onto the steel surface, thereby strengthening the protective film. Collectively, the insights from MEP mapping and Mulliken charge distributions provide compelling molecular-level evidence for the superior adsorption behavior and inhibition efficiency of the studied ILs.

The Electron Localization Function (ELF) analysis, visualized through isosurfaces and a color-filled XY-plane map Fig. 15c and d, distinctly identifies the specific electron localization sites governing donor interactions. This mapping reveals a region of high electron density, distinctly marked in red, precisely localized around the coordinating atoms O13, O14, N15, and N16 in both inhibitors, indicating a specialized reactive center optimized for efficient electron transfer. In contrast, the surrounding molecular expanse is characterized by low electron localization, a property consistently evidenced by the characteristic color transition from green to blue across the planar maps^76,77^.

- Site-Specific Reactivity: Fukui Indices and Dual Descriptors.

Fukui function analysis was employed to probe the adsorption behavior of the studied inhibitors on the CSs, thereby offering deeper theoretical insights into the experimentally observed inhibition performance. This approach enables the identification of the most reactive atomic sites by distinguishing regions prone to nucleophilic and electrophilic attacks.

The condensed Fukui indices (f_K_^+^ for nucleophilic attack and f_K_⁻ for electrophilic attack), together with the local electrophilicity indices (ω_K_^+^, ω_K_⁻) and local softness parameters (σ_k_⁺, σ_k_⁻), were calculated and summarized in the Supplementary Tables. To allow systematic comparison across different atomic sites, differential parameters Δfₖ, Δσₖ, and Δωₖ were derived, defined as (f_K_ ^+^ − f_K_^⁻^), (σ_k_⁺ − σ_k_⁻), and (ω_K_^+^ − ω_K_^⁻^), respectively^41^. Positive values of these descriptors (Δf_K_, Δσ_k_, Δω_K_ > 0) denote regions more susceptible to nucleophilic attack, while negative values (Δf_K_, Δσ_k_, Δω_K_ < 0) highlight atomic centers favorable for electrophilic attack.

**Fig. 17:**
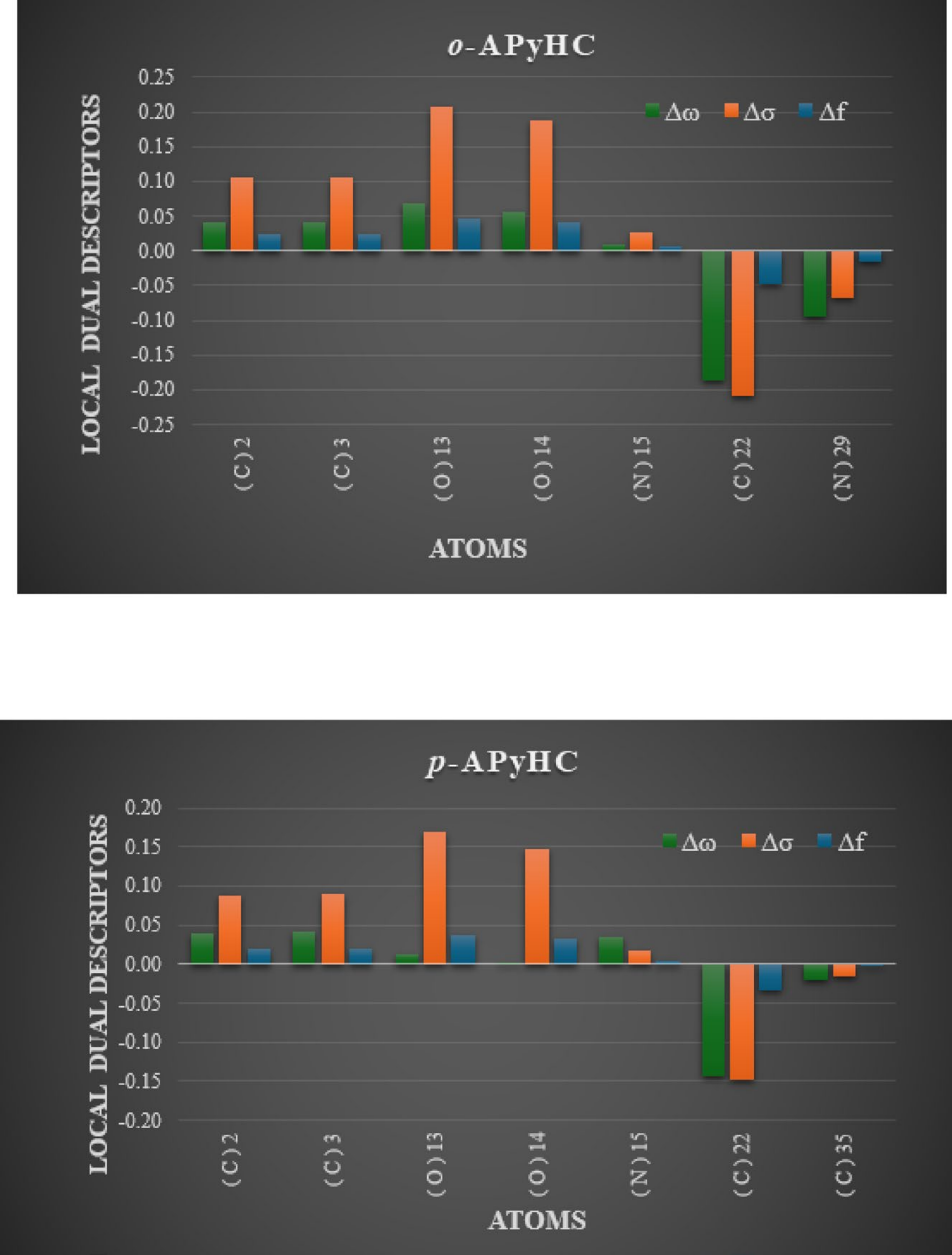
Local dual descriptors of different atoms with respect to Mulliken charges of **APyHC**.

According to these calculations, nucleophilic reactivity was predominantly localized on atoms C2, C3, O13, O14, and N15 in both *p*-**APyHC** and *o*-**APyHC**, indicating these atoms as key donor sites for interaction with the steel surface. Conversely, electrophilic susceptibility was observed at C22 and C35 in *p*-**APyHC** and at C22 and N29 in *o*-**APyHC**. The identification of these distinct reactive centers provides a molecular-level explanation of the adsorption mechanism, confirming that the presence of multiple nucleophilic sites in *p-APyHC* significantly enhances its capacity to donate electrons to the metal surface, thereby rationalizing its superior inhibition efficiency compared to *o-APyHC* (Fig. 17). Based on Fukui analysis and frontier molecular orbital results, the adsorption process between the investigated inhibitor and the carbon surface is most accurately described as a donor-acceptor interaction.

### Molecular docking insights into antibacterial activity

**Fig. 18:**
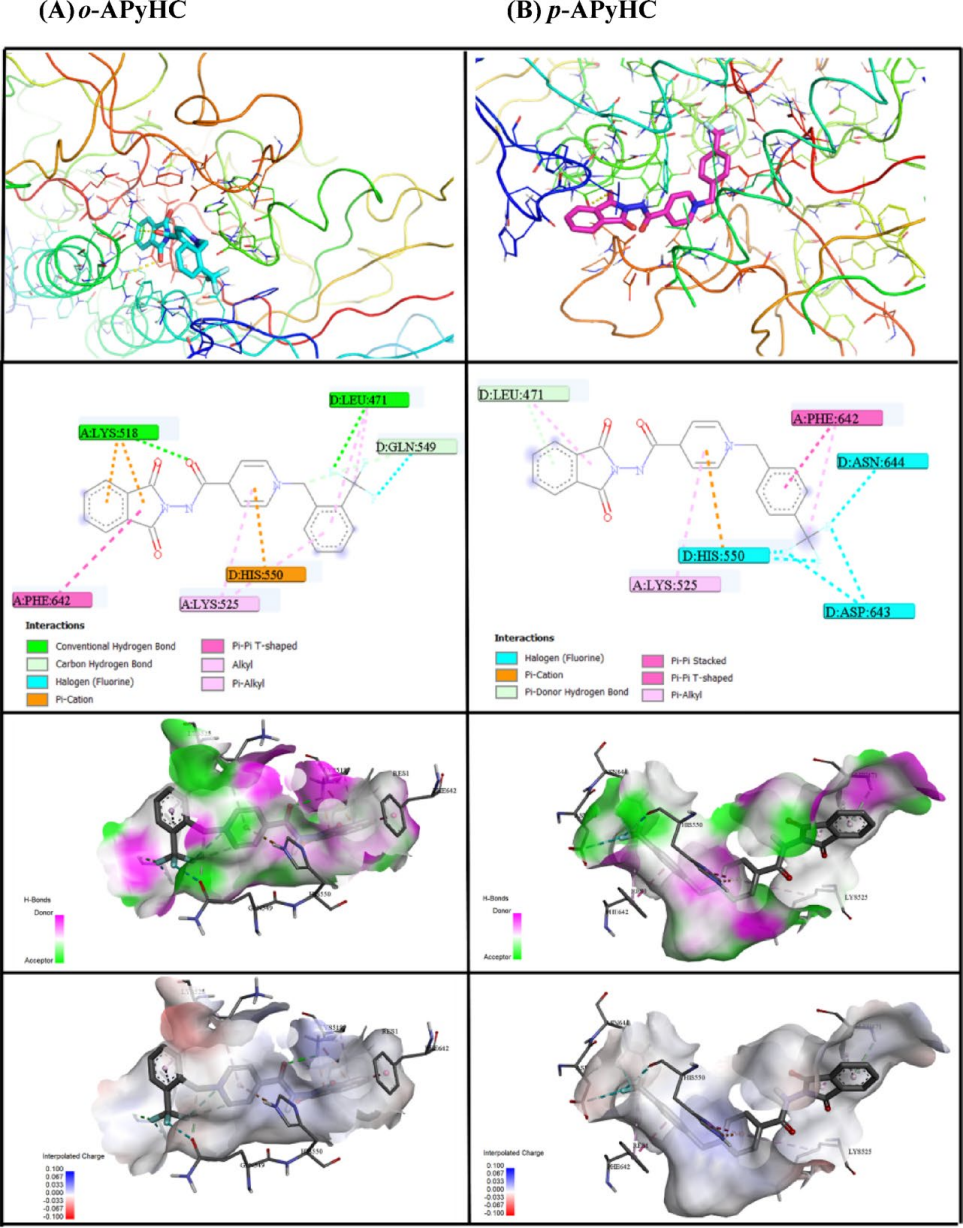
2D and 3D docking interactions of (*o*-**APyHC**) and (*p*-**APyHC**) in the active pocket of ribosyl transferase (PDB ID: 3GEY), visualized with Discovery Studio software.

The molecular docking results (Fig. 18; Table 9) demonstrated that both *o-APyHC *and *p-APyHC* exhibited strong and stable binding to the ribosyl transferase active site, highlighting their potential as effective antibacterial candidates^78^. The binding energies were − 10.2 kcal/mol for *o-APyHC* and − 10.4 kcal/mol for *p-APyHC*, with corresponding inhibition constants of 36.94 nM and 26.41 nM, confirming the efficiency of both ligands in occupying the catalytic pocket and preventing substrate access^79^. *o-APyHC* formed multiple stabilizing interactions, including two strong hydrogen bonds with LYS518 (2.77 Å) and LEU471 (2.66 Å), a carbon–hydrogen bond (3.18 Å), and three halogen interactions involving F44, F45, and F46. Additional stabilization arose from π-cation interactions with LYS518:NZ (3.80 Å) and HIS550:NE2 (4.65 Å), together with π-alkyl and π–π interactions involving PHE642, LEU471, LYS518, and LYS525. Similarly, *p-APYHC* displayed an even stronger affinity (–10.4 kcal/mol), forming a key hydrogen bond with LEU471 (3.20 Å) and five significant halogen interactions, particularly with ASN644 (2.91 Å) and ASP643 (3.36–3.59 Å), which played a crucial role in stabilizing the complex. π–cation interactions with HIS550 (4.80 Å), π–π stacking with PHE642 (3.63 Å), and multiple hydrophobic contacts further enhanced the ligand’s stability within the active site. Since ribosyltransferase is essential for RNA/DNA biosynthesis in bacteria, the strong binding of both ligands disrupts nucleic-acid precursor formation, inhibits bacterial growth, and prevents cell damage. Overall, the dense network of hydrogen bonds, halogen contacts, electrostatic interactions, and hydrophobic packing confirms the capability of both ligands to effectively block the enzyme, with *p-APyHC* showing slightly superior inhibitory characteristics and representing a promising candidate for further antibacterial development.

**Fig. 19:**
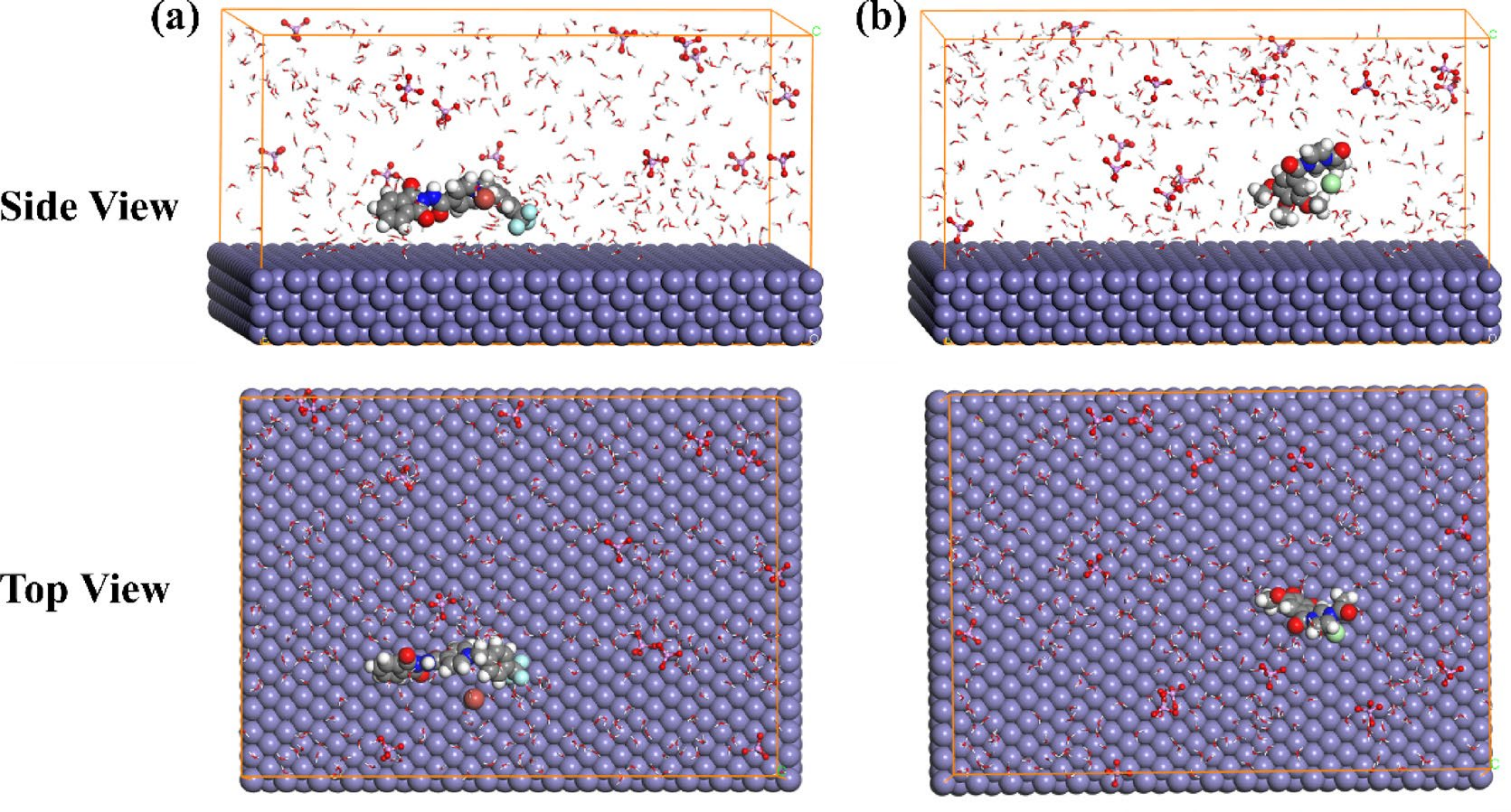
Adsorption configurations of *p-APyHC* and *o-APyHC*inhibitor molecules on the Fe (110) surface in H₃PO₄ medium, showing top and side views obtained from molecular dynamics (MD) simulations.

**Table 9:** Molecular docking scores for *o*-**APyHC** and *o*-**APyHC**, binding energies, inhibition constant, and Protein–Ligand interaction of the synthesized molecules in the active site of ribosyl transferase (PDB ID: 3GEY).

ILs	Binding Energy (ΔG) Kcal/mol	Inhibition Constant (K_i_) nM	Protein–Ligand interaction and bond distance (Å)
*o*-APYHC	−10.2	36.94	LYS518―O19 (Hydrogen Bond-2.77 Å) LEU471―F44 (Hydrogen Bond-2.66 Å) RES1―F45 (Carbon H-bond-3.18 Å) LYS518―π-system (π–Cation-3.80 Å) HIS550―π-system (π–Cation-4.65 Å)
*p*-APYHC	−10.4	26.41	LEU471―π-system (Hydrogen Bond-3.20 Å) ASN644―F44 (Halogen-2.91 Å) ASP643―F44 (Halogen-3.36 Å) ASP643―F46 (Halogen-3.59 Å) HIS550―π-system (π–Cation-3.63 Å) PHE642―C43 (π–allkyl-3.59 Å)

### Molecular dynamics simulations (MD)

Molecular dynamics (MD) simulations were further performed to provide a deeper molecular-level understanding of the adsorption behavior of the investigated inhibitors on the carbon steel surface in phosphoric acid medium. The simulation procedure was initiated by cleaving pure iron along the Fe (110) crystallographic plane, which is considered one of the most thermodynamically stable surfaces for adsorption studies. A simulation box was subsequently constructed containing the Fe (110) substrate together with inhibitor molecules, H_2_O molecules, and H_3_PO_4_ molecules to realistically mimic the corrosive environment. MD simulations represent an effective theoretical approach for investigating the adsorption mechanism and interaction strength between corrosion inhibitors and metallic surfaces^80^.

The optimized adsorption configurations of **p-APyHC** and **o-APyHC** on the Fe (110) surface are illustrated in Fig. 19. The obtained configurations reveal that both inhibitor molecules adsorb nearly parallel to the metal surface, maximizing the contact area between the inhibitor molecules and the Fe substrate. Such adsorption orientation facilitates strong interaction between the electron-rich adsorption centers, particularly nitrogen and oxygen heteroatoms, as well as π-electrons of the aromatic rings, and the vacant 3 d orbitals of iron atoms. Consequently, a compact and stable protective film forms over the metal surface, effectively blocking the penetration of aggressive, corrosive species such as H_3_PO_4_ molecules and thereby reducing corrosion^81^.

The adsorption behavior was quantitatively evaluated through the adsorption energy (E_ads_), which was calculated according to Eq. (9):

9 $$\:{E}_{ads}={E}_{Inhibitor/Metal}-\left({E}_{Inhibitor}+{E}_{Metal}\right)$$

where a more negative adsorption energy indicates stronger and more spontaneous adsorption of the inhibitor molecule onto the metal surface. The calculated adsorption energies were found to be − 846.55 kcal/mol for **p-APyHC** and − 816.88 kcal/mol for *o-*APyHC. The significantly more negative Eads value obtained for **p-APyHC** indicates a stronger interaction and higher adsorption affinity toward the Fe (110) surface than for **o-APyHC**. This result confirms that **p-APyHC** forms a more stable adsorbed layer on the steel surface, leading to superior corrosion inhibition efficiency. The MD simulation findings therefore agree well with the experimental measurements and DFT calculations, collectively confirming the enhanced inhibition performance of **p-APyHC** in a phosphoric acid medium.

### Proposed dissolution inhibition mechanism

**Fig. 20:**
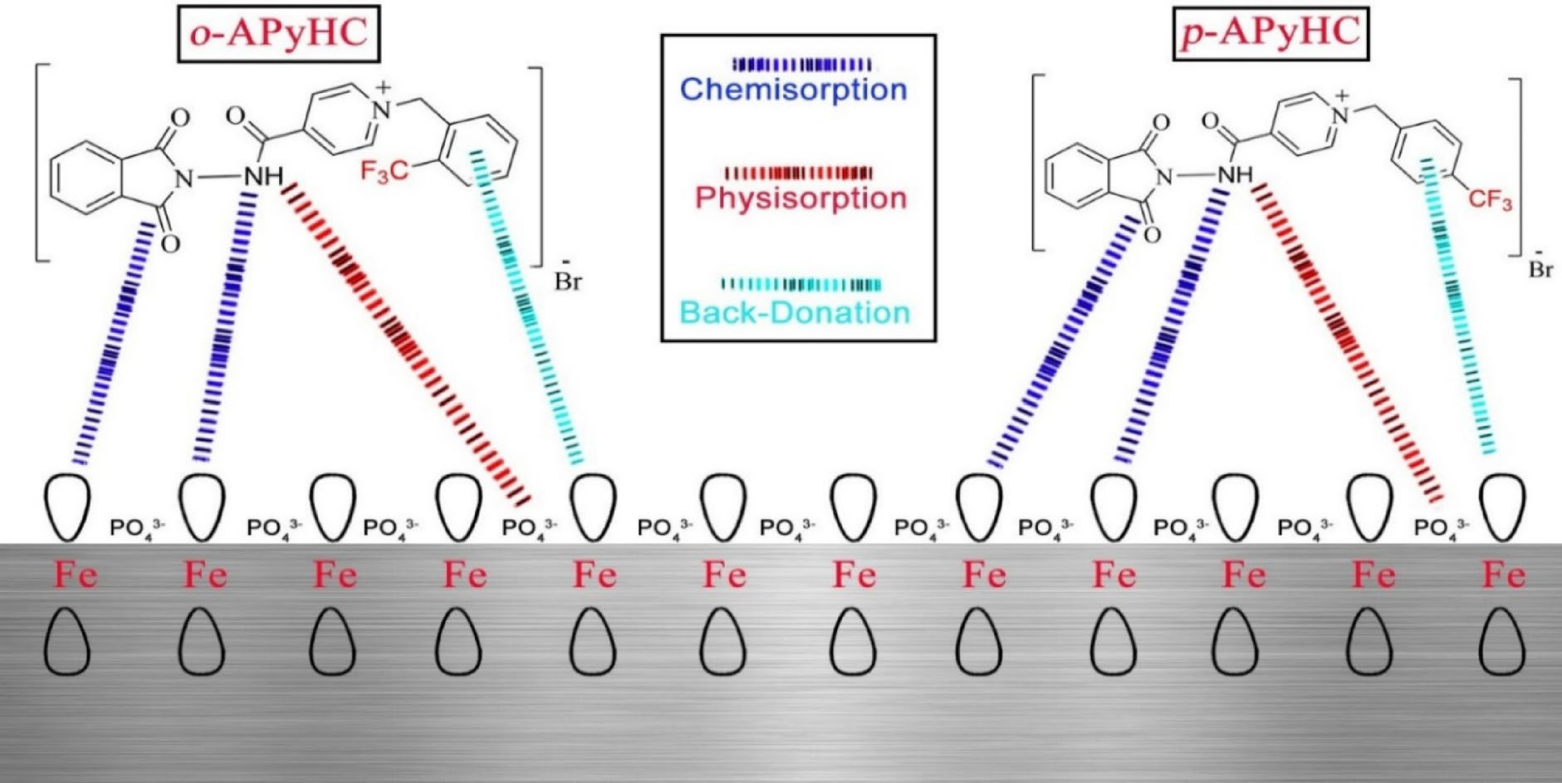
The proposed reaction mechanism that inhibits EP of CSs using **APyHC**.

In an acidic medium (Fig. 20), the CSs’surfaces acquires a positive charge^22^. This charge electrostatically attracts the anionic part $\:\ddot{NH}.$ However, the presence of CF_3_ in the ortho position could proinhibit the close intermolecular interaction due to the steric hindrance. The resulting coordinate complex analog is due to the interaction between the $\:\ddot{NH}$ IL cation moity that could promote the physicochemical adsorption of *p-APyHC* compound. The physical nature of this interaction is corroborated by the negative values of the fractional number of electrons transferred (ΔN), indicating electron donation by the inhibitor.

Concurrently, the calculated free energy of adsorption (ΔG°_ads_) values confirm that chemisorption operates alongside physisorption^82^. This chemical adsorption occurs via dual electron-transfer processes:

1. Electron Donation: Lone pairs from heteroatoms *O*,* N*, and π-electrons from aromatic rings are donated into vacant d-orbitals of surface iron atoms^83^.
2. Back-Donation: Electrons from the 3 d orbitals of iron may be transferred to the vacant antibonding molecular orbitals (π*) of the inhibitor^45^.

The synergy of this donation and back-donation results in a stable, protective film on the metal surface^53^. Therefore, it is confirmed that the isoniazid-based ionic liquids function via a combined physisorption-chemisorption mechanism, effectively inhibiting both corrosion and wear.

### Comparative analysis with pyridinium salt inhibitors

The controlled metal dissolution required for processes like electropolishing relies on the use of acidic media in industry. To achieve the desired surface finish and uniformity, specific inhibitors are needed to modulate the dissolution rate. The *pyridinium salt* family of organic compounds has proven particularly effective for this role. A comparison of the two aromatic *pyridinium salt-based isonized* molecules investigated in this work with other reported derivatives is provided in Table 10. These compounds, as numerous studies emphasize, demonstrate excellent performance in regulating metal dissolution, as validated by multiple experimental and computational approaches.

### The environmental compatibility and safety evaluation of a pyridinium-modified isoniazid compound

This research describes the transformation of isoniazid into pyridinium salt derivatives, aiming to improve its therapeutic properties while actively reducing its environmental impact. The approach aligns with sustainable pharmacy by designing these ionic derivatives to enhance biodegradability and reduce ecotoxicity to non-target organisms, potentially decreasing the drug’s persistence and harm to ecosystems. Furthermore, the increased bioavailability of the salt form could lower the required dosage per patient, thereby reducing the quantity of the active ingredient excreted into the environment. The research also encompasses the application of green chemistry principles in the synthesis process and a detailed study of the compound’s behavior in aquatic systems, collectively embodying a proactive, “benign-by-design” strategy to address pharmaceutical pollution without compromising clinical efficacy^86,87,88^.

**Table 10:** Comparative inhibition efficiencies of pyridinium salt inhibitors reported in literature and the present work under electropolishing conditions. .

Inhibitor	Concentration	Metal/Medium	Technique	Inhibition efficiency (%)	Reference
BPH	(50–100) ppm	C-steel/15% HCl	Potentiodynamic polarization	51.8–84.6	^18^
[C_4_Py] Br	(0.25–8) mmol L^− 1^	MS/1 M HCl	Potentiodynamic Polarization	70.1–90.4	^84^
Py12	(0.5–15) mmol dm^− 3^	EN3B mild steel/3.5% NaCl solution	Tafel Polarization	68.92–76.92	^85^
*o*-APyHC	(4.94*10^− 6^−5.928*10^− 5^) M	C-steel/8M H_3_PO_4_	Galvanostatic Polarization	46.67–74.67	Current Work
*p-*APyHC	(4.94*10^− 6^−5.928*10^− 5^) M	C-steel/8M H_3_PO_4_	Galvanostatic Polarization	53.33–80	Current Work

## Conclusion and future outlook

- This study successfully developed and evaluated two novel aromatic pyridinium salt-based isoniazid hybrid compounds (**APyHC**) as eco-friendly ionic liquid inhibitors for the electropolishing of carbon steel in aggressive 8 M H₃PO₄. The synthesized **APyHC** inhibitors demonstrated high inhibition efficiencies, reaching 80.99% for **p-APyHC** and 74.67% for **o-APyHC** at a low concentration (5.93 × 10⁻⁵ M), showcasing their potential as sustainable alternatives to conventional toxic inhibitors.
- A combined experimental and computational approach was employed to elucidate the inhibition mechanism. Galvanostatic polarization studies revealed that APyHC adsorption follows a mixed physisorption-chemisorption mechanism, obeying the El-Awady isotherm model. Thermodynamic parameters confirmed spontaneous and endothermic adsorption, with ΔG°_ads_ values ranging from − 37.40 to −39.77 kJ/mol. Surface characterization via SEM-EDX, AFM, and XPS confirmed the formation of a protective film that significantly reduced surface roughness and corrosion product formation.
- DFT calculations provided atomistic insights into the reactivity and adsorption behavior of the inhibitors, highlighting the superior electron-donating ability and reactivity of *p-***APyHC**compared to *o*-**APyHC**.
- The molecular docking results demonstrate that both *o*-**APyHC** and ***p*APyHC** exhibit strong and stable binding affinities for ribosyltransferase, indicating a promising inhibitory mechanism against bacterial nucleic acid biosynthesis. The high binding energies and nanomolar inhibition constants (Ki) underscore their potential as effective antibacterial lead compounds.
- Future investigations should focus on in vitro antibacterial activity and enzyme inhibition assays to validate the computational predictions experimentally. Additionally, further structural optimization and comprehensive biological evaluation are recommended to enhance inhibitory efficiency and assess their feasibility for practical antimicrobial application.
- Overall, this work presents a green, efficient, and multifunctional ionic liquid-based inhibitor system that aligns with sustainable industrial practices and offers promising applications in both material protection and biological inhibition.

## Supplementary Information

Below is the link to the electronic supplementary material.

Supplementary Material 1

## Data Availability

The data used and analyzed during the current study are available from the corresponding authors upon reasonable request.

## References

[1] Abbott, A. et al. Electrolytic metal coatings and metal finishing using ionic liquids. *ECS Trans.* **16**, 47. 10.1149/1.3114008 (2009).

[2] Melia, M. A. et al. How build angle and post-processing impact roughness and corrosion of additively manufactured 316L stainless steel. *npj Mater. Degrad.* **4**, 21. 10.1038/s41529-020-00126-5 (2020).

[3] Lebedeva, O. et al. Advantages of electrochemical polishing of metals and alloys in ionic liquids. *Metals* **11**, 959. 10.3390/met11060959 (2021).

[4] Widyanto, B. et al. Optimization of the Effect of Electropolishing’s Current Density and Time on Roughness, Microstructure, and Corrosion Resistance. *J. Energy Mech. Mater. Manuf. Eng.* **6**, 197. 10.22219/jemmme.v6i3.19828 (2021).

[5] Buettner, C. S. et al. Surface-active ionic liquids: A review. *J. Mol. Liq.* **347**, 118160. 10.1016/j.molliq.2021.118160 (2022).

[6] Modwi, M. M. Y. et al. Eco-friendly corrosion inhibitor of Q235 carbon steel in 1.0 M HCl by Isatin/Chitosan Schiff base. *J. Mol. Struct.* **1321**, 139592. 10.1016/j.molstruc.2024.139592 (2025).

[7] Parangusan, H. et al. Plant extract as green corrosion inhibitors for carbon steel substrate in different environments: A systematic review. *Int. J. Electrochem. Sci.* **20**, 100919. 10.1016/j.ijoes.2024.100919 (2025).

[8] Souza, L. et al. Ionic liquids as corrosion inhibitors for carbon steel protection in hydrochloric acid solution: A first review. *J. Mater. Res. Technol.* **22**, 2186. 10.1016/j.jmrt.2022.12.066 (2023).

[9] Othman, K. A. et al. Theoretical and experimental exploration of organic molecules adsorption on iron surfaces for corrosion inhibition: A review. *Corros. Rev.* **43**, 335. 10.1515/corrrev-2024-0039 (2025).

[10] Ghehsareh, Z. et al. Green guardians: A comprehensive review of environmentally friendly corrosion inhibitors from plant extract to ionic-liquids in industrial applications. *Prog. Org. Coat.* **201**, 109146. 10.1016/j.porgcoat.2025.109146 (2025).

[11] Fu, S. et al. Investigating the effect of pyridine substituent position on the corrosion inhibition performance of novel benzophenone derivatives: Experimental and theoretical study. *J. Environ. Chem. Eng.* **13**, 116909. 10.1016/j.jece.2025.116909 (2025).

[12] Guendouz, A. et al. New benzimidazole derivatives as efficient organic inhibitors of mild steel corrosion in hydrochloric acid medium: Electrochemical, SEM/EDX, MC, and DFT studies. *J. Mol. Struct.* **1321**, 139901. 10.1016/j.molstruc.2024.139901 (2025).

[13] Toghan, A. et al. Unraveling adsorption and interaction of synthesized chlorinated cyclic imide derivative on the corrosion of C-Steel in H2SO4 solution: Experimental control and mathematical calculations. *ACS Omega* **10**, 46593. 10.1021/acsomega.5c03599 (2025). 41114211 10.1021/acsomega.5c03599 PMC12529203

[14] Ait Mansour, A. et al. Insights into the corrosion inhibition performance of isonicotinohydrazide derivatives for N80 steel in 15% HCl medium: An experimental and molecular level characterization. *Metals* **13**, 797. 10.3390/met13040797 (2023).

[15] Reis, C. L. B. et al. Crystallization of isoniazid in choline-based ionic liquids: Physicochemical properties and anti-tuberculosis activity. *J. Mol. Liq.* **394**, 123671. 10.1016/j.molliq.2023.123671 (2024).

[16] Uddin, E. et al. Biological applications of isoniazid-derived Schiff base complexes: an overview. *Asian J. Res. Biochem.* **6**, 17. 10.9734/AJRB/2020/v6i330118 (2020).

[17] Chaouiki, A. et al. Inhibitory effect of a new isoniazid derivative as an effective inhibitor for mild steel corrosion in 1.0 M HCl: combined experimental and computational study. *Res. Chem. Intermed.* **46**, 2919. 10.1007/s11164-020-04119-6 (2020).

[18] Assad, H. et al. Evaluating the adsorption and corrosion inhibition capabilities of Pyridinium - P - Toluene Sulphonate on MS in 1 M HCl medium: An experimental and theoretical study. *Inorg. Chem. Commun.* **153**, 110817. 10.1016/j.inoche.2023.110817 (2023).

[19] Abbott, A. P. et al. Anodic dissolution of metals in ionic liquids. *Progress Nat. Science: Mater. Int.* **25**, 595. 10.1016/j.pnsc.2015.11.005 (2015).

[20] Singh, A. et al. Evaluation of corrosion mitigation properties of pyridinium-based ionic liquids on carbon steel in 15% HCl under the hydrodynamic condition: Experimental, surface, and computational approaches. *J. Mol. Liq.* **376**, 121408. 10.1016/j.molliq.2023.121408 (2023).

[21] Ikeuba, A. I. et al. Anti-corrosion performance of ethyl dimethyl propylammonium bis (trifluoromethyl sulfonyl) imide (EDMPA-TFSI) for AA3003 alloy in acid chloride solutions in the presence and absence of potassium iodide. *J. Ionic Liquids*. **4**, 100099. 10.1016/j.jil.2024.100099 (2024).

[22] Aslam, R. et al. Bio-based ionic liquid as a corrosion inhibitor for mild steel in 5% HCl solution: Experimental and theoretical investigation. *Sustainable Chem. Pharm.* **39**, 101614. 10.1016/j.scp.2024.101614 (2024).

[23] Sowbhagyam, D. V. Ionic liquids as green solvents: a comprehensive review. *Int. Res. J. Adv. Eng. Hub*. **2**, 220 (2024). https://irjaeh.com

[24] El-Sonbaty, A. A. et al. Corrosion Inhibitors, Molecular Docking and Quantum Chemical Parameters Of Allyl Rhodanine Azodye Derivatives and Developed To Protect C-Steel in Acidic Environments. *Egypt. J. Chem.* **68**, 407. 10.21608/ejchem.2024.284076.9618 (2025).

[25] Sayed, F. N. et al. Design, spectroscopic characterization, DFT, molecular docking, and different applications: Anti-corrosion and antioxidant of novel metal complexes derived from ofloxacin-based Schiff base. *J. Organomet. Chem.* **993**, 122698. 10.1016/j.jorganchem.2023.122698 (2023).

[26] Motawea, M. M. et al. One potential method of recycling expired thiocolchicoside drug is to use an environmental corrosion inhibitor for carbon steel in HCl. *Sci. Rep.* **15**, 27799. 10.1038/s41598-025-07609-y (2025). 40738915 10.1038/s41598-025-07609-y PMC12310934

[27] Remache, S. et al. Mechanistic understanding and performance assessment of *Urospermum dalechampii*-based green inhibitors for enhanced corrosion resistance of carbon steel in acidic media. *Colloids Surf. A. Physicochem. Eng. Asp.* **727**, 138076. 10.1016/j.colsurfa.2025.138076 (2025).

[28] Taha, A. A. et al. Synthesis and evaluation of nonionic surfactants based on dimethylaminoethylamine: Electrochemical investigation and theoretical modeling as inhibitors during electropolishing in ortho-phosphoric acid. *J. Mol. Liq.* **328**, 115421. 10.1016/j.molliq.2021.115421 (2021).

[29] Fahim, A. M. et al. Numerous heterocyclic compounds with an isonicotinic moiety have been studied for their synthesis, antibacterial, anticancer, docking simulation, and DFT characteristics. *Polycycl. Aromat. Compd.* **44**, 5707. 10.1080/10406638.2023.2266549 (2024).

[30] Moustafa, A. H. E. et al. Mass transfer role in electropolishing of carbon steel in H_3_PO_4_ containing amino acids: Electrochemical, computational, SEM/EDX, and stylus profilometer investigation. *Alex. Eng. J.* **61**, 6305. 10.1016/j.aej.2021.11.062 (2022).

[31] Kotabag, S. D. et al. Chemical and electrochemical investigation of poly (vinyl alcohol) cobalt nanoparticles composite as corrosion inhibitor for soft-cast steel in 1 M HCl medium. *Surf. Interfaces* **74**, 107689. 10.1016/j.surfin.2025.107689 (2025).

[32] Ettahiri, W. et al. Synthesis, characterization, theoretical, and evaluation of eco-friendly phenytoin-based corrosion inhibitors for mild steel. *Colloids Surf. A. Physicochem. Eng. Asp.* **707**, 135816. 10.1016/j.colsurfa.2024.135816 (2025).

[33] Zgueni, Hea et al. Synthesis, structural characterization, and inhibition effects of new bicatenar surfactants based of phenolphtalein on the corrosion of carbon steel in 1 M HCl: Experimental and computational insights. *J. Mol. Liq.* **127116**, 127116. 10.1016/j.molliq.2025.127116 (2025).

[34] Li, EeaC. et al. Combined electrochemical, surface analysis, and DFT/MD simulations to evaluate choline histidine ionic liquid as a green corrosion inhibitor for mild steel in neutral medium. *Materials Today Communications* **109871**, 109871. 10.1016/j.mtcomm.2024.109871 (2024).

[35] Fawzy, A. et al. Inhibition evaluation of chromotrope dyes for the corrosion of mild steel in an acidic environment: Thermodynamic and kinetic aspects. *ACS Omega* **6**, 4051. 10.1021/acsomega.0c06121 (2021). 33585780 10.1021/acsomega.0c06121 PMC7876844

[36] Hamza, et al. Eco-friendly corrosion inhibitor chitosan methionine for carbon steel in 1 M hydrochloric acid solution: Experimental and theoretical approach. *Sci. Rep.* **2**, 15924. 10.1038/s41598-025-98981-2 (2025). 10.1038/s41598-025-98981-2 PMC12059160 40335621

[37] Toghan, et al. Inhibition effects of citrulline and glutamine for mild steel corrosion in sulfuric acid environment: Thermodynamic and kinetic aspects. *International Journal of Electrochemical Science* **40**, 211118. 10.20964/2021.11.40 (2021).

[38] Motawea, et al. Fabrication of protective organic layer using thiazolyl-pyrazolone compound as an efficient corrosion inhibitor for CS-N80 in 0.5 M H_2_SO_4_ solution. *J. Mol. Struct.* **141469**, 141469. 10.1016/j.molstruc.2025.141469 (2025).

[39] Keshar, et al. Corrosion inhibition and adsorption behavior of benzothiazole compounds on mild steel in an acidic environment: Experimental and theoretical approach. *Colloids and Surfaces A: Physicochemical and Engineering Aspects* **136318**, 136318. 10.1016/j.colsurfa.2025.136318 (2025).

[40] Toghan, A. et al. Adsorption mechanism, kinetics, thermodynamics, and anticorrosion performance of a new Thiophene derivative for C-Steel in a 1.0 M HCl: Experimental and computational approaches. *Metals* **13**, 1565. 10.3390/met13091565 (2023).

[41] Moustafa, A. H. E. et al. Anticorrosive performance of newly synthesized dipyridine-based ionic liquids by experimental and theoretical approaches. *Sci. Rep.* **13**, 19197. 10.1038/s41598-023-45822-9 (2023). 37932361 10.1038/s41598-023-45822-9 PMC10628253

[42] Bouklah, M. et al. Effect of substitution on corrosion inhibition properties of three Imidazole derivatives on mild steel in 1 M HCl. *Arab. J. Chem. Environ. Researches*. **7**, 126–143 (2020). www.mocedes.org/ajcer

[43] Adamu, A. et al. Adsorption and thermodynamic studies of the corrosion Inhibition effect of Desmodium adscendens (Swartz) extract on carbon steel in 2 M HCl. *BMC Chem.* **19**, 163. 10.1186/s13065-025-01541-y (2025). 40490798 10.1186/s13065-025-01541-y PMC12147248

[44] Mahmmod, A. et al. On the adsorption isotherms behavior of quinoaxaline as corrosion inhibitor for copper in nitric acid. *Moroccan J. Chem.* **13**, 495. 10.48317/IMIST.PRSM/morjchem-v13i2.54884 (2025).

[45] Zheng, X. et al. Experimental and theoretical studies of two imidazolium-based ionic liquids as inhibitors for mild steel in sulfuric acid solution. *Corros. Sci.* **95**, 168. 10.1016/j.corsci.2015.03.012 (2015).

[46] Yousif, Q. A. et al. High-performance corrosion inhibitors for carbon steel in hydrochloric acid: electrochemical and DFT studies. *RSC Adv.* **15**, 28666. 10.1039/D5RA04952K (2025). 40861989 10.1039/d5ra04952k PMC12377231

[47] Shathani, P. et al. Thermodynamics and adsorption behavior of Sclerocarya birrea leaf extract as a potential green corrosion inhibitor for mild steel in a simulated seawater (3.5% NaCl) environment. *Chem. Thermodyn. Therm. Anal.* **19**, 100197. 10.1016/j.ctta.2025.100197 (2025).

[48] Singh, A. K. Understanding of corrosion inhibition behavior of expired isoniazid modified benzothiazole 2-carboxaldehyde at mild steel/0.5 M H_2_SO_4_ interface. *Mater. Chem. Phys.* **319**, 129323. 10.1016/j.matchemphys.2024.129323 (2024).

[49] Vaszilcsin, C. G. et al. On the evaluation of metal-corrosion inhibitor interactions by adsorption isotherms. *J. Mol. Struct.* **1286**, 135643. 10.1016/j.molstruc.2023.135643 (2023).

[50] Fawzy, A. et al. Thermodynamic, kinetic, and mechanistic approach to the corrosion inhibition of carbon steel by new synthesized amino acids-based surfactants as green inhibitors in neutral and alkaline aqueous media. *J. Mol. Liq.* **265**, 276. 10.1016/j.molliq.2018.05.140 (2018).

[51] Shi, F.-F. et al. Synergistic inhibition of 5,6-Diamino-1,10-Phenanthroline with chloride ion on mild steel corrosion in 5.5 M H_3_PO_4_ containing 2% H_2_SO_4_ solution. *Int. J. Electrochem. Sci.* **17**, 221290. 10.20964/2022.12.80 (2022).

[52] Abbas, M. A. et al. Adsorption, thermodynamic, and quantum chemical investigations of an ionic liquid that inhibits corrosion of carbon steel in chloride solutions. *Sci. Rep.* **12**, 12536. 10.1038/s41598-022-16755-6 (2022). 35869239 10.1038/s41598-022-16755-6 PMC9307760

[53] El-Nagar, R. A. et al. Evaluation of ionic liquids based imidazolium salts as an environmentally friendly corrosion inhibitors for carbon steel in HCl solutions. *Sci. Rep.* **14**, 1889. 10.1038/s41598-024-52174-5 (2024). 38253588 10.1038/s41598-024-52174-5 PMC10803315

[54] Eissa, M. E. et al. Sweet Orange Peel Extract as green sustainable corrosion inhibitor for Al in 1 M HCl. *Int. J. Electrochem. Sci.* **20**, 100882. 10.1016/j.ijoes.2024.100882 (2025).

[55] Abdelshafi, N. S. et al. In-depth experimental assessment of two new aminocoumarin derivatives as corrosion inhibitors for carbon steel in HCl media combined with AFM, SEM/EDX, contact angle, and DFT/MDs simulations. *J. Mol. Struct.* **1304**, 137638. 10.1016/j.molstruc.2024.137638 (2024).

[56] Modwi, M. M. Y. et al. A novel chitosan Schiff base derivatives as eco-friendly corrosion inhibitors for Q235 carbon Steel: A combined experimental and theoretical study. *J. Phys. Chem. Solids*. **208**, 113205. 10.1016/j.jpcs.2025.113205 (2025).

[57] Pour-Ali, S. & Hejazi,. Tiazofurin drug as a new and non-toxic corrosion inhibitor for mild steel in HCl solution: Experimental and quantum chemical investigations. *J. Mol. Liq.* **118886**, 118886. 10.1016/j.molliq.2022.118886 (2022).

[58] Zheng, T. et al. Synergistic corrosion inhibition effects of quaternary ammonium salt cationic surfactants and thiourea on Q235 steel in sulfuric acid: Experimental and theoretical research. *Corros. Sci.* **199**, 110199. 10.1016/j.corsci.2022.110199 (2022).

[59] Qiu, L. et al. Comparison of the corrosion inhibition property on cold-rolled steel in sulfuric acid media between reflux and ultrasound extracts from rapeseed meal. *Ind. Crops Prod.* **216**, 118809. 10.1016/j.indcrop.2024.118809 (2024).

[60] Peng, Y. et al. Adsorption behavior and inhibition performance of octadecyl dimethyl benzyl ammonium chloride on steel surface in phosphoric acid medium: Experimental and theoretical investigations. *Chin. J. Chem. Eng.* **83**, 72. 10.1016/j.cjche.2025.03.016 (2025).

[61] Zhang, Q. et al. Chitosan derivatives as promising green corrosion inhibitors for carbon steel in acidic environment: Inhibition performance and interfacial adsorption mechanism. *J. Colloid Interface Sci.* **640**, 1052. 10.1016/j.jcis.2023.02.141 (2023). 36921384 10.1016/j.jcis.2023.02.141

[62] Varatharajan, P. et al. Hydrothermal synthesis of orange fluorescent carbon dots and their application in fabrication of warm WLEDs and fluorescent ink. *Physica B* **654**, 414703. 10.1016/j.physb.2023.414703 (2023).

[63] Kumari, P. et al. Synthesis of novel carbon dots as efficient green corrosion inhibitor for mild steel in an acidic environment: Electrochemical, gravimetric, and XPS analysis. *Prog. Org. Coat.* **209**, 109561. 10.1016/j.porgcoat.2025.109561 (2025).

[64] Wang, H. et al. Synergistic mixture of *Eupatorium adenophora* spreng leaves extract and KI as a novel green inhibitor for steel corrosion in 5.0 M H_3_PO_4_. *J. Mater. Res. Technol.* **23**, 5082. 10.1016/j.jmrt.2023.02.160 (2023).

[65] El Faydy, M. et al. Electrochemical, surface, and computational studies on the inhibition performance of some newly synthesized 8-hydroxyquinoline derivatives containing benzimidazole moiety against the corrosion of carbon steel in phosphoric acid environment. *J. Mater. Res. Technol.* **9**, 727. 10.1016/j.jmrt.2019.11.014 (2020).

[66] Zaidon, F. H. et al. Adsorption and corrosion inhibition accomplishment for thiosemicarbazone derivatives for mild steel in 1.0 M HCl medium: Electrochemical, XPS, and DFT studies. *J. Mol. Liq.* **329**, 115553. 10.1016/j.molliq.2021.115553 (2021).

[67] Khamaysa, O. M. A. et al. Hydrazone-based green corrosion inhibitors for API grade carbon steel in HCl: Insights from electrochemical, XPS, and computational studies. *Colloids Surf. A Physicochem. Eng. Aspects* **626**, 127047. 10.1016/j.colsurfa.2021.127047 (2021).

[68] Abdelsalam, M. M. et al. Green synthesis, electrochemical, and DFT studies on the corrosion inhibition of steel by some novel triazole Schiff base derivatives in hydrochloric acid solution. *Arab. J. Chem.* **15**, 103491. 10.1016/j.arabjc.2021.103491 (2022).

[69] Hsissou, R. Review on epoxy polymers and its composites as a potential anticorrosive coatings for carbon steel in 3.5% NaCl solution: Computational approaches. *J. Mol. Liq.* **336**, 116307. 10.1016/j.molliq.2021.116307 (2021).

[70] Abdallah, M. et al. Insight of corrosion mitigation performance of SABIC iron in 0.5 M HCl solution by tryptophan and histidine: Experimental and computational approaches. *Int. J. Hydrogen Energy* **47**, 12782. 10.1016/j.ijhydene.2022.02.007 (2022).

[71] El-Azabawy, O. E. et al. Studying the temperature influence on carbon steel in sour petroleum media using facilely-designed Schiff base polymers as corrosion inhibitors. *J. Mol. Struct.* **1275**, 134518. 10.1016/j.molstruc.2022.134518 (2023).

[72] Tran, H. H. et al. Structures, electronic properties, and interactions of Cetyl Alcohol with Cetomacrogol and water: Insights from quantum chemical calculations and experimental investigations. *American Chemical Society Omega (ACS Omega)* **6**, 20975. 10.1021/acsomega.1c02439 (2021). 34423205 10.1021/acsomega.1c02439 PMC8374918

[73] Dutta, A. et al. Correlating electronic structure with corrosion inhibition potentiality of some bis-benzimidazole derivatives for mild steel in hydrochloric acid: Combined experimental and theoretical studies. *Corros. Sci.* **98**, 541. 10.1016/j.corsci.2015.05.065 (2015).

[74] Abd-El-Nabey, BAeaTaa. et al. Trizma as an eco-friendly, efficient inhibitor for the acidic corrosion of steel: Experimental and computational studies. *Sci. Rep.* **4**, 15346. 10.1038/s41598-022-19060-4 (2022). 10.1038/s41598-022-19060-4 PMC9468175 36097017

[75] Saady, AeaM. et al. Molecular dynamics, DFT, and electrochemical to study the interfacial adsorption behavior of new imidazo[4,5-b] pyridine derivative as corrosion inhibitor in acid medium. *J. Appl. Electrochem.* **51**, 245. 10.1007/s10800-020-01498-x (2021).

[76] Emara, M. M. et al. Electronic and structural perturbations of microporous ZIF-67 nanoparticles and Cr(VI) molecule during adsorptive water decontamination unveiled by experimental and quantum computational investigations. *J. Mol. Liq.* **390**, 123042. 10.1016/j.molliq.2023.123042 (2023).

[77] Li, et al. A surface molecular assembly-based composite inhibitor for mitigating corrosion in dynamic supercritical CO2 aqueous environment. *Chem. Eng. J.* **153193**, 153193. 10.1016/j.cej.2024.153193 (2024).

[78] Mandour, HSeaEc. et al. Exploring corrosion behavior, antimicrobial evaluation, molecular docking, and DFT calculation of thiosemicarbazone ligand and its metal complexes. *Sci. Rep.* **1**, 16577. 10.1038/s41598-025-98580-1 (2025). 10.1038/s41598-025-98580-1 PMC12075617 40360666

[79] Hassan, EMeaNf. et al. Nanoformulations for hydrazones; synthesis, characterization, parasitology, and histopathology investigations. *ACS Omega* **10**, 20226. 10.1021/acsomega.4c10965 (2025). 40454056 10.1021/acsomega.4c10965 PMC12120574

[80] Fouad, M. et al. Mn–Co-BTC@ MOF/S-MXene composite with superior efficiency for Pb (II) ion removal: Mechanistic and DFT study. *Chemosphere* **385**, 144526. 10.1016/j.chemosphere.2025.144526 (2025). 40553970 10.1016/j.chemosphere.2025.144526

[81] Al-Qurashi, O. S. & Wazzan,. Molecular and periodic DFT calculations of the corrosion protection of Fe (1 1 0) by individual components of *Aerva lanata* flower as a green corrosion inhibitor. *J. Saudi Chem. Soc.* **101566**, 101566. 10.1016/j.jscs.2022.101566 (2022).

[82] Hajjaji, F. E. et al. Pyridinium-based ionic liquids as novel eco-friendly corrosion inhibitors for mild steel in molar hydrochloric acid: Experimental & computational approach. *Surf. Interfaces* **22**, 100881. 10.1016/j.surfin.2020.100881 (2021).

[83] Alharbi, M. et al. Exploring corrosion protection of mild steel by ionic liquid functionalized graphene oxide: Gravimetric, electrochemical, and surface studies. *Results Chem.* **13**, 101985. 10.1016/j.rechem.2024.101985 (2025).

[84] Luo, F. et al. Alkyl pyridine ionic liquid as a green corrosion inhibitor for mild steel in acidic medium. *RSC Adv.* **15**, 27369. 10.1039/D5RA04148A (2025). 40757155 10.1039/d5ra04148a PMC12314875

[85] Talat, R. et al. Evaluating the corrosion inhibition efficiency of pyridinium-based cationic surfactants for EN3B mild steel in acidic-chloride media. *Coatings* **12**, 1701. 10.3390/coatings12111701 (2022).

[86] Anjaneyulu, B. et al. Toward sustainable chemistry: A survey of green synthesis methods for pyridine derivatives (A review). *Chem. Biodivers.* **22**, e202403328. 10.1002/cbdv.202403328 (2025). 39966092 10.1002/cbdv.202403328

[87] Borikhonov, B. et al. Development of new sustainable pyridinium ionic liquids: From reactivity studies to mechanism-based activity predictions. *J. Mol. Model.* **30**, 359. 10.1007/s00894-024-06157-y (2024). 39356293 10.1007/s00894-024-06157-y

[88] Rezki, N. et al. An eco-friendly ultrasound-assisted synthesis of novel fluorinated pyridinium salts-based hydrazones and antimicrobial and antitumor screening. *Int. J. Mol. Sci.* **17**, 766. 10.3390/ijms17050766 (2016). 27213367 10.3390/ijms17050766 PMC4881586

