# Supplementary material for: Isoniazid-derived ionic liquids for enhanced C-steels surface finishing via electrochemistry, supported by Monte Carlo, mechanistic study, theoretical, and docking methodology
Source: Sci Rep. 2026 Jun 16;16:18665. doi: 10.1038/s41598-026-55745-w (PMC13272921; doi:10.1038/s41598-026-55745-w)
Supplement: Supplementary file 1 — Supplementary Material 1 [file 41598_2026_55745_MOESM1_ESM.docx]

**Isoniazid-Derived Ionic Liquids for Enhanced C-steels Surface Finishing via Electrochemistry, Supported by Monte Carlo, Mechanistic Study, Theoretical, and Docking Methodology**

*Amira Hossam Eldin Moustafa, Ali Atya Harfoush, Hanaa Hammam Abdel-Rahman, Mohamed Hagar, Menna Mamdouh*

Chemistry Department, Faculty of Science, Alexandria University, P.O. 426 Ibrahemia, Alexandria 21321, Egypt.

- **Materials and Equipment**

All reactions were carried out in oven-dried glassware under anhydrous conditions. NMR spectra were recorded on a JEOL JNM-ECA spectrometer operating at 500 MHz for ¹H NMR and 125 MHz for ¹³C NMR, using deuterated solvents as both the deuterium lock and internal reference. ¹³C NMR spectra were acquired using the UDEFT pulse sequence with broadband proton decoupling. Chemical shifts (δ) are reported in parts per million (ppm) relative to tetramethylsilane (TMS) as an internal standard. Fourier-transform infrared (FT-IR) spectroscopy was performed on a Bruker Tensor 37 spectrometer using potassium bromide (KBr) pellets, with characteristic absorption bands reported as wavenumbers (ν_max, cm⁻¹).

**Sup.Fig 1**: FT-IR of ***o*-APyHC**


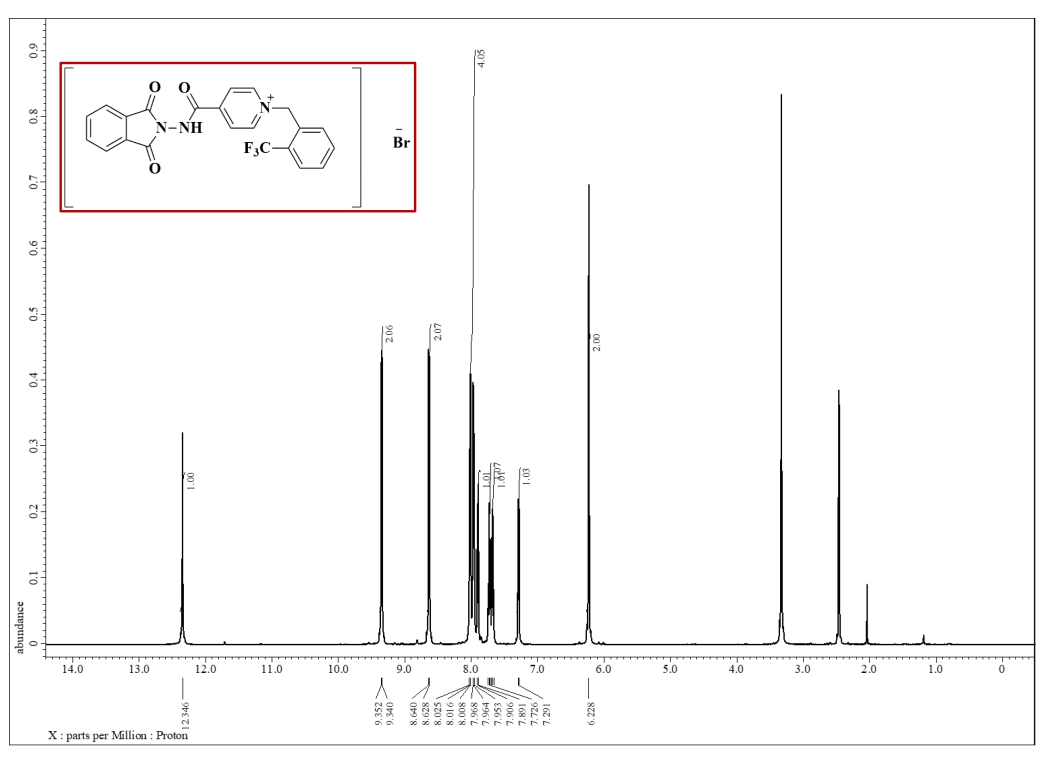

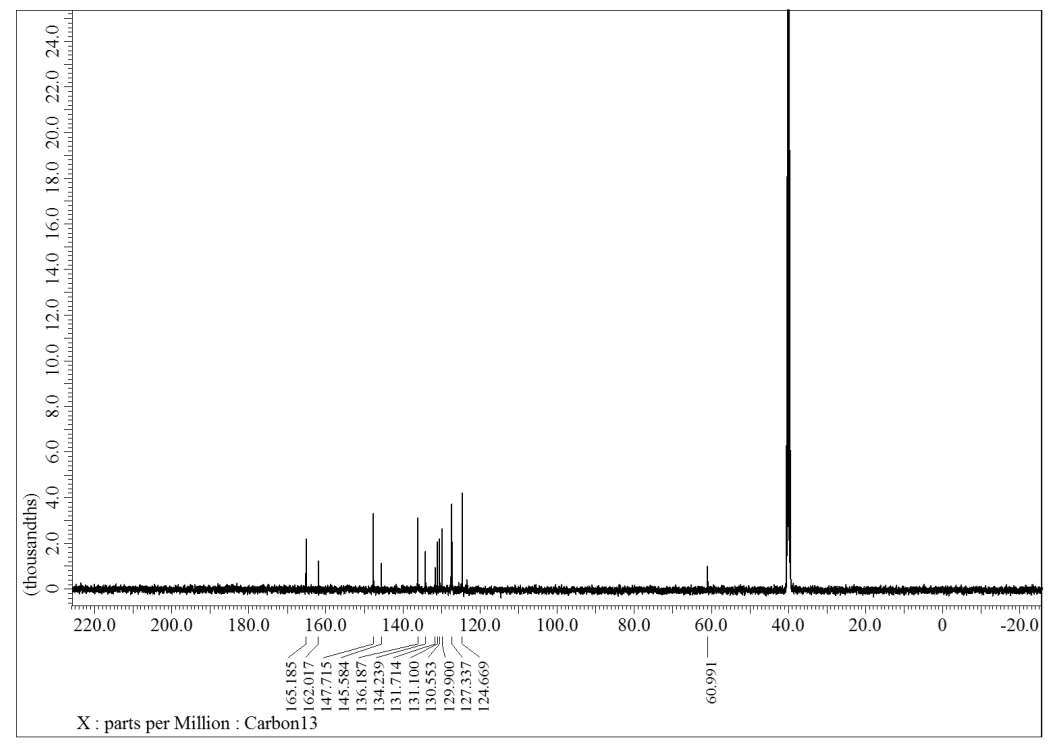
**Sup.Fig 2**: ^1^ H NMR of ***o*-APyHC**

**Sup.Fig 3**: C^13^ NMR of ***o*-APyHC**

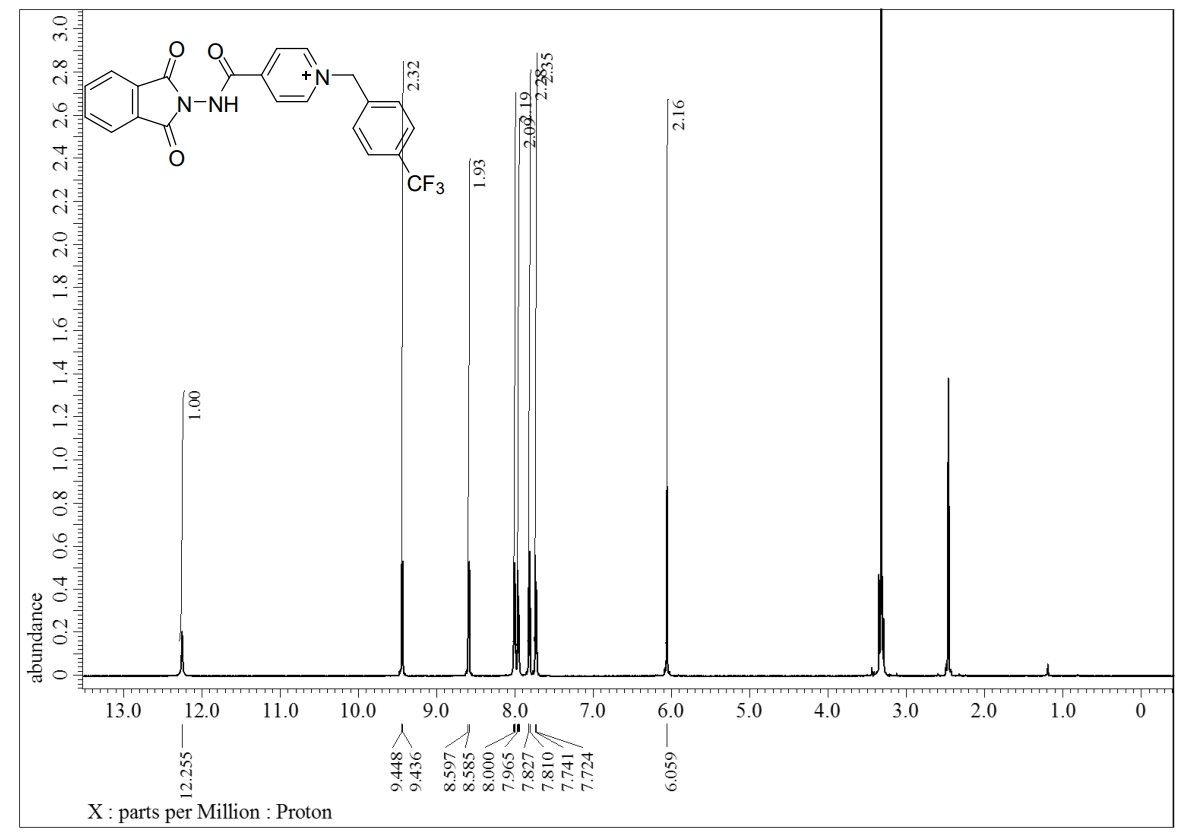
**Sup.Fig 4**: FT-IR of ***p*-APyHC**

**Sup.Fig 5**: ^1^ H NMR of ***p*-APyHC**


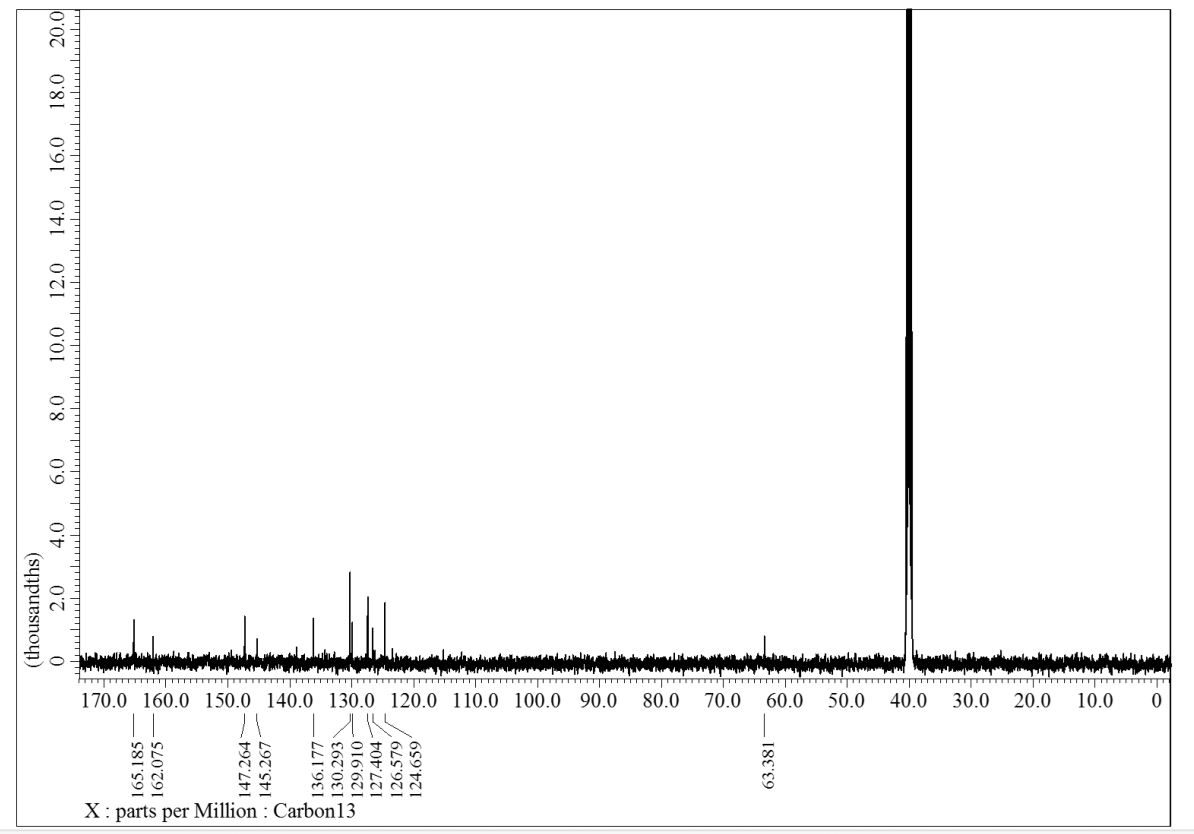


**Sup.Fig 6**: C^13^ NMR of ***p*-APyHC**

**Sup. Table 1:** Equations for evaluating quantum chemical descriptors

| **I =** $\mathbf{-}$ **E_HOMO_** | **A =** $\mathbf{-}$ **E_LUMO_** | **X =**$\frac{\mathbf{(I}\boldsymbol{+}\mathbf{A)}}{\boldsymbol{2}}$ |
| --- | --- | --- |
| **P_i_ =** $\mathbf{-}$ **X** | **ƞ =** $\frac{\mathbf{(I} \mathbf{- A)}}{\boldsymbol{2}}$ | **σ =**$\frac{\boldsymbol{1}}{\mathbf{ƞ}}$ |
| **∆E_b.d_** $\mathbf{=}\frac{\mathbf{-ƞ}}{\boldsymbol{4}}$ | **ω =** $\frac{\boldsymbol{P}_{\boldsymbol{i}}^{\boldsymbol{2}}}{\mathbf{2ƞ}}$ | **ω^-^ =** $\frac{\mathbf{(3I+A)}^{\mathbf{2}}}{\mathbf{16(I-A)}}$ |
| **ω^+^ =** $\frac{\mathbf{(I+3A)}^{\mathbf{2}}}{\mathbf{16(I-A)}}$ | **∆ω^±^ = (ω^+^- (-ω^-^))** | **∆ω^±^ = (ω^+^-** $\frac{\mathbf{1}}{\boldsymbol{\omega}^{\mathbf{-}}}$**)** |
| **∆N =** $\frac{\left( \mathbf{X}_{\mathbf{Fe}}\mathbf{- X} \right)}{\mathbf{2}\left( \mathbf{ƞ}_{\mathbf{Fe}}\mathbf{+ ƞ} \right)}$ | **∆E_steel/inh_ =** $\frac{\left( \mathbf{X}_{\mathbf{Fe}}\mathbf{-}\mathbf{X}_{\mathbf{inh}} \right)^{\boldsymbol{2}}}{\boldsymbol{4}\left( \mathbf{ƞ}_{\mathbf{Fe}}\mathbf{+}\mathbf{ƞ}_{\boldsymbol{inh}} \right)}$ | |

**Sup.Table 2**: A comprehensive analysis of **APyHC**: Mulliken charges, Fukui functions, and the dual descriptor.
